# Genetic diversity within *Strongyloides fuelleborni*: mitochondrial genome analysis reveals a clear African and Asian division

**DOI:** 10.1017/S0031182025100243

**Published:** 2025-06

**Authors:** Travis Richins, Sarah G. H. Sapp, Alexandra Juhasz, Lucas J. Cunningham, E. James La Course, J. Russell Stothard, Joel L. N. Barratt

**Affiliations:** 1 Division of Parasitic Diseases and Malaria, Laboratory Sciences and Diagnostic Branch, Centers for Disease Control and Prevention, Atlanta, GA, USA; 2 Department of Tropical Disease Biology, Liverpool School of Tropical Medicine, Liverpool, UK; 3 Microbiology Department, Semmelweis University, Budapest, Hungary, UK

**Keywords:** Africa, Asia, paraphyly, population structure, *Strongyloides fuelleborni*, strongyloidiasis

## Abstract

Following the recent report of strongyloidiasis caused by *Strongyloides fuelleborni* within a semi-captive colony of baboons in a UK safari park, we investigated the genetic relationships of this isolate with other *Strongyloides* isolates across the world. Whole-genome sequencing data were generated with later phylogenetic analysis of mitochondrial (mt) cytochrome oxidase subunit 1 (*cox1*) and nuclear ribosomal 18S sequences against 300 published *Strongyloides* reference genotypes. The putative African origin of the UK *S. fuelleborni* was confirmed and full-length mt genome sequences were assembled to facilitate a more detailed phylogenetic analysis of 14 mt coding regions against all available *Strongyloides* species. Our analyses demonstrated that the UK isolate represented a novel African lineage not previously described. Additional complete mt genomes were assembled for several individual UK safari park worms to reveal a slightly altered mt genome gene arrangement, allowing clear separation from Asian *S. fuelleborni*. Furthermore, these UK worms possessed expanded intergenic regions of unknown function that increase their mt genome size to approximately 24 kilobases (kb) as compared with some 16 kb for Asian *S. fuelleborni*; this may have arisen from unique populational founder and genetic drift effects set within the peculiar mixed species baboon and drill ancestry of this semi-captive primate colony. A maximum likelihood phylogeny constructed from 14 mt coding regions also supported an evolutionary distinction between Asian and African *S. fuelleborni*.

## Graphical abstract

**Figure:**
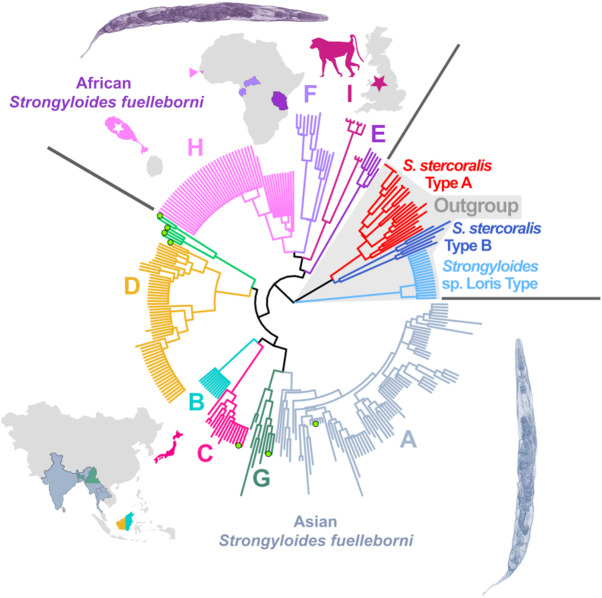

## Introduction

Human strongyloidiasis is an important helminthiasis caused by *Strongyloides stercoralis* and sometimes *Strongyloides fuelleborni*, the latter species is particularly common in various Old World non-human primates. These threadworm nematodes each have unique and complex life cycles transitioning between parasitic and free-living states, and between sexual and asexual stages, with sex occurring exclusively in the environment (Hunt et al., 2016; Buonfrate et al., 2024). *Strongyloides stercoralis* and *S. fuelleborni* are distinct in certain aspects of their parasitic life cycle and some clinical aspects. Female parthenogenic *S. stercoralis* deposit eggs in the intestinal mucosa that hatch to release rhabditiform larvae that enter the intestinal lumen and are passed in stool. Larvae may also penetrate the intestinal wall to enter the circulation, perpetuating an auto-infectious cycle that can lead to life-threatening hyper-infections (Nutman, 2017). Alternatively, *S. fuelleborni* parthenogenic females lay eggs that enter the lumen and are passed in stool, hatching in the environment, yielding rhabditiform larvae. Thus, rhabditiform larvae in stool are diagnostic for *S. stercoralis* infection, and not *S. fuelleborni*, for which the presence of eggs in stool is diagnostic. As *S. fuelleborni* eggs hatch in the environment, autoinfection is absent.

Global interest in *S. fuelleborni* is increasing, partly because human infections with this species seem more common than previously thought, despite this threadworm being principally considered a non-human primate specialist (Cunningham et al., 2025). Of historical note is the taxonomy of these threadworms. The name *S. fuelleborni* von Linstow (1905) was originally reserved for *Strongyloides* infecting chimpanzees and yellow baboons in Africa while *Strongyloides simiae* Hung and Höppli (1923) was described from Asian macaques. The validity of *S. simiae* was questioned soon after its description, as differences forming the basis of its distinction from *S. fuelleborni* were deemed insufficient or too variable (Sandground, 1925; Little, 1966). This culminated in a report by Premvati (1959), synonymizing *S. simiae* with *S. fuelleborni*. Decades later, *S. fuelleborni* underwent further taxonomic adjustment when reports of infections caused by *S. fuelleborni*-like nematodes in infants from Papua New Guinea emerged (Viney et al., 1991; Ashford et al., 1992). The subspecies *S. fuelleborni kellyi* was erected to accommodate this novel Papuan *Strongyloides*; *S. fuelleborni* from old-world apes and monkeys was designated as the nominotypical subspecies *S. fuelleborni fuelleborni*.

Sequencing technologies afford new opportunities to scrutinize previously established taxonomic relationships among *Strongyloides* species, providing new knowledge on population-level genetic substructures (Barratt and Sapp, 2020; Buonfrate et al., 2024). This knowledge offers valuable insights from an epidemiologic perspective, including whether certain *Strongyloides* populations are human specialists (i.e., anthroponotic), rarely (or never) infect people, or are host-promiscuous and/or potentially zoonotic (Jaleta et al., 2017; Barratt and Sapp, 2020; Richins et al., 2023; de Ree et al., 2024). Recent molecular studies have revealed genetic sub-structuring among *S. f. fuelleborni*, with differences seemingly being driven by vicariance (Barratt and Sapp, 2020; Ko et al., 2023; Richins et al., 2023). However, studies supporting this African and Asian distinction are primarily based on only two genotyping loci: mitochondrial (mt) cytochrome oxidase subunit 1 (*cox1*) and nuclear ribosomal 18S rDNA (Hasegawa et al., 2010; Ko et al., 2023; Richins et al., 2023). Recently, Ko et al. (2023) constructed a phylogeny based on 12 mitochondrial protein-coding genes from Asian *S. fuelleborni* and various other *Strongyloides* species. This work provided valuable information on the evolutionary relationships between various *Strongyloides* species (Ko et al., 2023), although African *S. fuelleborni* were absent from that analysis simply due to a lack of genomic information.

Our present study sought to take advantage of the recent report of *S. fuelleborni* within a semi-captive colony of UK baboons (Juhasz et al., 2023) where fresh material of larval worms could be readily obtained. In so doing, we hoped to better characterize the diversity within this population of *S. fuelleborni*, which – to our knowledge – represents its most northerly occurrence across the world, and elucidate the genetic relationship between African and Asian *S. fuelleborni* strains with increased resolution. Whole-genome sequencing (WGS) data (Illumina) were generated from worms from this UK *S. fuelleborni* population with clustering of *cox1* and 18S sequences extracted from this WGS data alongside 300 published *Strongyloides* reference genotypes. After which, full-length mt genome sequences were assembled from several individual worms to facilitate a more detailed phylogenetic analysis of 14 mt coding regions from various *Strongyloides* species and several Asian isolates of *S. fuelleborni* previously sequenced by Ko et al. (2023).

## Materials and methods

### Available nematode material

**Figure 1.**
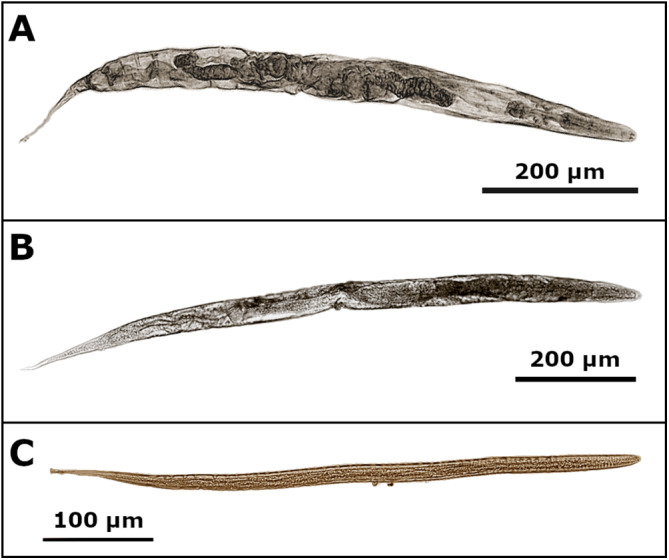
Examples of female adult (A and B) and filariform larvae (C) of UK *Strongyloides fuelleborni.* The figure provides examples of adult female and filariform larvae of *S. fuelleborni* cultured from UK baboons via the charcoal incubation method. The nematodes show clear evidence of desiccation and shrivelling due to long-term storage in 90% ethanol. Despite this, the transport and storage conditions (90% ethanol, ambient temperature) were sufficient to obtain enough genetic material from several specimens for whole-genome sequencing. Image taken on an Olympus BX41 microscope using Olympus CellSens software. Image was rendered performed using the GIMP [link].

Various larvae and adults of *S. fuelleborni* were obtained from faecal charcoal culture from sampled baboons within Knowsley Safari, in the northwest of England, Merseyside (53.4339° N, 2.8126° W) (Juhasz et al., 2023). Today, the colony of some 240 animals is comprised mostly of olive baboons (*Papio Anubis*), although historically the founding colony also consisted of a small mixture of hamadryas (*Papio hamadryas*), chacma (*Papio ursinus*), yellow baboon (*Papio cynocephalus*) and drill (*Mandrillus leuophaeus*) (Juhasz et al., 2023). The prevalence of strongyloidiasis within the colony is approximately 10% with the identity of worms initially ascertained upon inspection of nuclear ribosomal 18S sequences (accession numbers: OR395366 and OR395367), finding closest matches with *S. fuelleborni fuelleborni*, with 100% query coverage and 99.08% identity (accession number AB272235) (Juhasz et al., 2023). In Spring 2024, fresh faecal samples were obtained and subjected to charcoal culture incubation (Dancescu, 1968; Yelifari et al., 2005), and when observed, *Strongyloides* adults and/or larvae (Figure 1) were placed in 95% ethanol and sent at ambient temperature to the Division of Parasitic Diseases and Malaria, at the US Centers for Disease Control and Prevention (CDC) in Atlanta, Georgia. Upon arrival at the CDC, individual nematodes were selected from the ethanol suspension using a pipette and were transferred to a 2 mL Eppendorf tube (one per nematode) for subsequent genome sequencing.

### Whole-genome sequencing

DNA extraction was performed on several larvae and adults of *S. fuelleborni*. One DNA extraction was performed for each nematode so that each subsequent genome would represent one individual of *S. fuelleborni* (i.e., pooling was not performed). Eppendorf tubes containing single worms in ethanol were centrifuged at 300 *g* for 10 min to move the worm to the bottom of the tube. The supernatant was aspirated and DNA was extracted from worms using a QIAamp UCP DNA Micro Kit (Qiagen, USA) following the manufacturer’s instructions, with elution in 30 µL of Buffer EB. Genomic DNA concentration was determined using a Qubit dsDNA HS Assay Kit (Thermo Fisher, USA), and extracts were diluted to the same concentration in PCR-grade water. Diluted genomic DNA was subjected to library preparation using a Nextera XT DNA Library Prep Kit according to the manufacturer’s instructions (Illumina, USA). DNA concentration and molecular size were assessed for the resultant library using a Qubit dsDNA HS Assay Kit (Thermo Fisher) and a High Sensitivity D1000 ScreenTape on the 2200 TapeStation (Agilent, USA), respectively. The quantified library was diluted to 10–15 pM, and WGS was performed on the NextSeq 1000 platform using a NextSeq 1000/2000 P2 kit (300 cycles) (Illumina). WGS data were submitted to NCBI under BioProject PRJNA1254725 and BioSamples SAMN48121344–SAMN48121350.

### Constructing 18S and cox1 consensus sequences

*Cox1* and 18S haplotype characterization was performed for UK worms using a custom Geneious workflow (Geneious Prime, ver. 2022: https://www.geneious.com). Illumina reads were imported into Geneious for trimming using BBDuk2 (parameters: trimq = 15, minlength = 25, qin = 33, other parameters set to default). Haplotypes of 18S hypervariable region 1 (HVR-I), 18S hypervariable region IV (HVR-IV) and partial *cox1* sequences were extracted from the trimmed/filtered reads by mapping them to *S. fuelleborni* reference sequences from GenBank (GB) (Accession Numbers: HVR-I, OQ190415.1; HVR-IV, OQ190403.1; *cox1*, MK463928.1). Mapping was performed using the inbuilt Geneious Mapper applying the ‘Medium Sensitivity/Fast’ setting, and a mapping consensus sequence was generated. Consensus sequences were compared to published HVR-I, HVR-IV and *cox1* reference sequences as described earlier.

### Reference sequences of 18S and cox1

The *S. fuelleborni* and *S. stercoralis cox1* and 18S rDNA reference sequences utilized here include those from Richins et al. (2023) and from earlier publications (Frias et al., 2018; Barratt et al., 2019; Barratt and Sapp, 2020; Janwan et al., 2020; Ko et al., 2023). Eight genotypes (complete sets of HVR-I, HVR-IV and *cox1* sequences from individual worm specimens) described by de Ree et al. (2024) from *S. fuelleborni* infecting humans in Bangladesh were also included. A *cox1* sequence extracted from the mitochondrial genome of *S. stercoralis* reference strain PV001 (GB: NC_028624.1) was also included. These data were used to construct a large and comprehensive set of *Strongyloides* reference genotypes including 30 from *S. stercoralis*, 18 from a *Strongyloides* sp. infecting slow lorises (the latter 48 genotypes serving as an outgroup) and 252 *S. fuelleborni* genotypes from various African and Asian primates (Supplementary File S1, Tab A). These 300 reference genotypes had been sequenced at varying combinations of *cox1*, HVR-I and HVR-IV. To maximize the volume of reference data included in our analysis, we utilized a genetic distance computation method (i.e., Barratt’s heuristic) that facilitates clustering of genotypes sequenced at different but overlapping combinations of loci (see below).

### Assigning genotypes to UK worms

**Figure 2.**
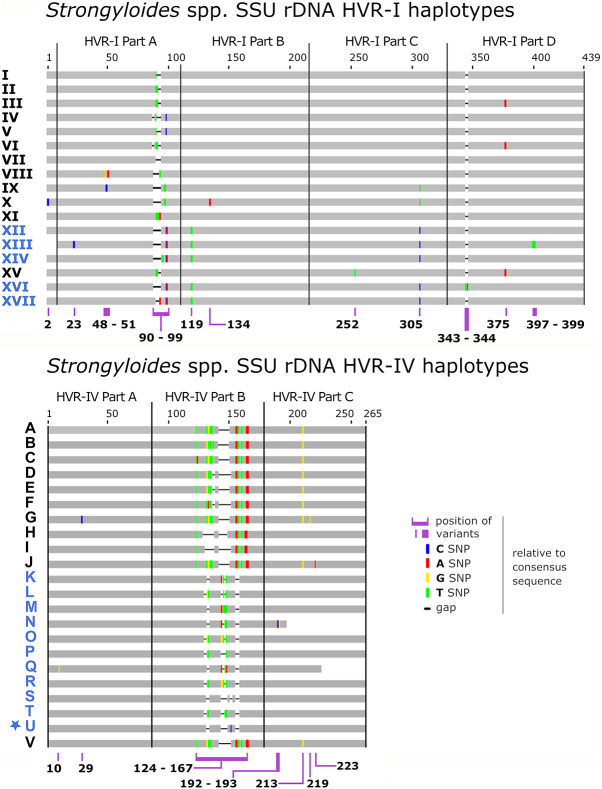
Schematic of the *Strongyloides* genotyping scheme referenced here. Graphical representation of an *Strongyloides* spp. 18S rDNA (or small subunit ribosomal DNA - SSU) genotyping scheme after earlier published descriptions (Barratt et al., 2019; Barratt & Sapp, 2020; Richins et al., 2023). This scheme was expanded here to include haplotype U of 18S HVR-IV (indicated by a star) identified here from captive baboons kept in Knowsley Safari (GenBank accessions in Supplementary Table S3). Haplotype names shown in blue belong to *S. fuelleborni* and those shown in black belong to other *Strongyloides* species in accordance with this typing scheme. Haplotype sequences are provided in Supplementary File S2. The figure also includes HVR-IV haplotype V identified by de Ree et al. (2024) in *S. stercoralis*.

Reference sequences were divided into segments for the characterization of haplotypes within each segment, as per Richins et al. (2023). HVR-I and HVR-IV segments are defined in Figure 2, while for *cox1*, reference sequence NC_028624.1 was divided into 15-mer segments ranging from A1, A2 and A3 to R1 (52 segments total). Only segments I1 through O1 (19 segments) were examined here, for reasons discussed elsewhere (Richins et al., 2023). *Cox1*, HVR-I and HVR-IV consensus sequences from UK worms were subjected to BLASTN searches against all *Strongyloides* reference sequences (Figure 2, Supplementary File S1, Appendix D). Haplotypes were assigned to UK worms following a 100% BLASTN match to a known haplotype. If a novel haplotype was identified, it was assigned a new sequential haplotype number as described (Richins et al., 2023). BLASTN results were used to construct a haplotype data sheet (HDS): a condensed format for representing haplotype data (Supplementary File S1, Tab B) and the required input for genetic distance computation using Barratt’s heuristic (Barratt et al., 2021; Jacobson et al., 2022).

### Genetic distance computation and clustering

Genetic distances were computed using the HDS generated from reference and UK worms, via the scripts available here: https://github.com/Joel-Barratt/Eukaryotyping. Notably, when using this method, a set of ‘minimum marker requirements’ must be defined (Supplementary File S2, Appendix A). As per Richins et al. (2023), genotypes had to possess a sequence for at least any 14 of the 19 *cox1* segments I1 through O1, to be included in this analysis. Genetic distances would thus be computed for genotypes with or without 18S data, so long as any 14 of these 19 *cox1* segments had been sequenced. Using this schema, resultant tree structures would be based mostly on *cox1*, though would also be influenced by HVR-I and/or HVR-IV information, where available (Barratt and Sapp, 2020). The resultant pairwise matrix (Supplementary File S1, Tab C) was clustered via the ‘agnes’ R package using Ward’s method (Ward, 1963) to generate a hierarchical tree. A neighbour-joining tree (Saitou and Nei, 1987) was generated from the same distance matrix using the ‘NJ’ function in the ‘ape’ R package. The ‘root’ function in the ‘ape’ package was used to root the tree at the *S. stercoralis*/*Strongyloides* sp. ‘Loris’ clade. The ‘ggtree’ R package was used to render trees. Host images were obtained from PhyloPic (https://www.phylopic.org/) or rendered in-house for tree annotation. Maps were generated in R using ggplot. Images were rendered using the GNU Image manipulation program (GIMP).

### Mitochondrial (mt) genome assembly and annotation

Trimmed and filtered WGS reads were aligned to an *S. fuelleborni* reference sequence (GB: OL672153) using the Geneious read mapper, applying the Medium Sensitivity/Fast mapping setting to generate a consensus sequence (Consensus #1). The same trimmed/filtered reads were next analysed using an inbuilt Geneious workflow that combines read mapping and *de novo* assembly, guided by Consensus #1 to generate a new contig (Contig #1). The trimmed/filtered reads were mapped to Contig #1 using the Geneious mapper, applying custom sensitivity parameters that allowed for zero alignment gaps, a minimum mapping similarity of 95% and no tolerance for ambiguous bases. The resultant alignment was manually examined for coverage and mapping accuracy within the Geneious interface. If poor mapping at certain regions was observed (e.g., sharp drops in coverage at any region), an issue with the assembly was assumed, and a second consensus (Consensus #2) was generated from this alignment. The combined mapping/*de novo* assembly process was repeated again guided by Consensus #2. Reads were mapped to this next assembly to assess it for accuracy by manual examination. This process was repeated until an accurate assembly was obtained. To annotate the assembly, coding regions were identified by homology to annotated genes within sequence OL672153. For tRNA genes, assembled Mt genomes were also examined using tRNAscan-SE 2.0 (Lowe and Chan, 2016).

### Mt gene phylogeny

Mt genomes from several *Strongyloides* spp. were downloaded from GB (accessions in Supplementary File S1, Table S1). Fourteen genes, including the mitochondrial 12S and 16S rRNA genes, plus 12 protein-coding genes (i.e., excluding any tRNA genes), were extracted from each Mt genome and concatenated for the construction of a phylogeny. The sequences were aligned using MAFFT in the Geneious interface (default parameters), and poorly aligned regions were realigned using MUSCLE with manual curation of alignments as required. Genes extracted from the mt genomes of *Parastrongyloides trichosuri* (GB: LC050209) and *Rhabditophanes* sp. KR302 (GB: LK995736.1) were included as an outgroup. The alignment was exported in fasta format for subsequent import into R via the read.phyDat function (phangorn R package). Genetic distances were computed using the dist.ml function (default parameters) and a neighbour-joining tree was constructed using the NJ function. The pml and modelTest functions were used to identify the best model for tree construction, where a model with the lowest Akaike information criterion and Bayesian information criterion values was considered optimal. The optim.pml function was used to optimize model parameters with the rearrangement parameter set to NNI (nearest neighbour interchange). The bootstrap.pml function was used to bootstrap the tree with 1000 replicates.

## Results

## UK S. fuelleborni represents a novel African population

A complete genotype (including HVR-I, HVR-IV and *cox1*) was obtained for nine UK worms and a partial genotype was obtained for four worms (Table 1). The nine worms with a complete genotype were clustered alongside the 300 reference genotypes. A complete mt genome was obtained from seven UK worms (Table 1). All UK worms possessed a novel HVR-IV haplotype (Table 2, Haplotype U). Haplotype XII of HVR-I, which is specific to African *S. fuelleborni* (Table 2), was detected in all UK worms thus confirming their putative African ancestry.

**Table 1.** Summary of UK *Strongyloides fuelleborni* genotypes

Specimen number	*Cox1* GenBank accession*	HVR-I	**HVR-IV**^a^
Filariform_larvae_76	PV564809	XII	U
Adult_01A^b^	PV555681	XII	U^c^
Adult_02A	PV564803	XII	U
Adult_05A	PV564804	XII	U
Adult_06A	PV564805	XII	U
Adult_07A	PV564806	XII	U
Adult_09A	PV564807	XII	U
Adult_10A	PV564808	XII	U
Adult_14A^b^	PV555680	XII	U
Filariform_larvae_06FL^b^	–	XII	U
Filariform_larvae_07FL^b^	–	–	U
Filariform_larvae_10FL^b^	–	XII	U
Filariform_larvae_31FL^b^	–	XII	U

*Note*: A *cox1* sequence was not obtained for any of the filariform larvae.

a Genotypes of UK *S. fuelleborni* possessed haplotype U: a novel HVR-IV haplotype identified here (GB: PV558842).

b Complete mt genome sequences were not obtained for these samples due to insufficient coverage.

c Sequence was truncated by 37 bases due to low coverage at one end but is otherwise identical to haplotype U.

* Accession numbers are provided for complete mitochondrial genome sequences, which contain the *cox1* gene.

**Table 2.** HVR-I and HVR-IV haplotypes of *Strongyloides fuelleborni*

Haplotypes	GenBank accession/s	Hosts
**HVR-I haplotypes of *S. fuelleborni***		
XII^b^	AB453320.1, AB453321.1, AB821045.1, AB821046.1, MN076377 – MN076380, OQ190413	Gorillas, chimpanzees, humans, baboons and vervets
XIII	AB453322.1	Gorillas
XIV	AB677955.1, AB272235.1, AB453317.1, AB453318.1, AB453319.1	Japanese macaques and humans^a^
XVI	OQ190414	Vervets
XVII	OQ190415	Vervets
**HVR-IV haplotypes of *S. fuelleborni***		
K	LC085496.1, LC085493.1, LC085492.1, LC085491.1, LC085484.1, LC085494.1, AB526820.1, AB453320.1, AB526823.1, AB526821.1	Chimpanzees and humans
L	LC085490.1, LC085489.1, OQ190398	Gorillas and vervets
M	LC085486.1, MN076407, OQ190400	Humans and vervets
N	LC085497.1	Chimpanzees
O	AB453322.1, OQ190401	Gorillas and vervets
P	LC085488.1, AB526824.1, AB526825.1, OQ190402	Chimpanzees, gorillas and vervets
Q	AB526822.1	Baboons
R	AB453321.1, OQ190403	Chimpanzees, vervets
S	KY081222.1, AB453317.1, AB453318.1, AB453319.1, AB272235.1, MH045486.1, MH045487.1, MH045487.1, MN076415, MT484268, MT484269	Humans, long-tailed macaques and pig-tailed macaques
T	MN076408, OQ190399	Human and vervets
U^c^	PV558842	UK baboons (Knowsley Safari)

*Note*: Haplotype XV of HVR-I was identified in an earlier study (Barratt and Sapp, 2020) and is intentionally excluded from this table as that haplotype belongs to *S. stercoralis* (table only shows *S. fuelleborni* haplotypes).

a No GB accession is provided for the detection of haplotype XIV in *S. fuelleborni* infecting humans; this refers to an observation reported by de Ree et al. (2024) who detected worms with this haplotype in humans from Bangladesh.

b HVR-I haplotype XII was previously identified in various African primates and was also identified in UK worms.

c Novel haplotype detected here from UK baboons in Knowsley Safari.

### Clustering and mt genome analysis

**Figure 3.**
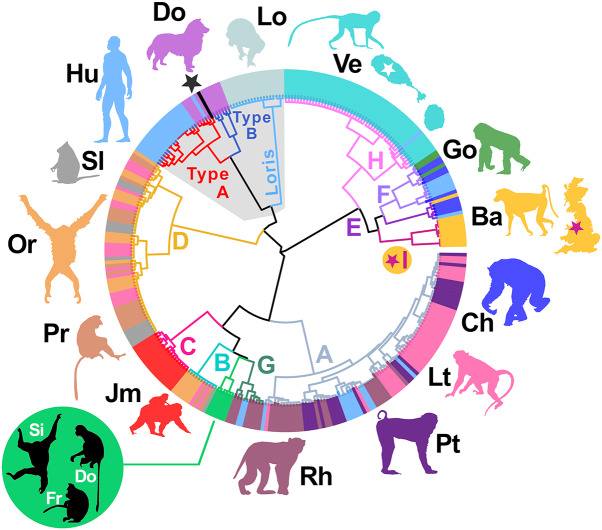
Hierarchical tree generated from *Strongyloides* reference genotypes and UK worms. This unrooted tree was generated using Ward’s method to cluster a pairwise distance matrix computed for 309 *Strongyloides* genotypes constructed from available *cox1*, HVR-I and HVR-IV sequences, including the nine genotypes from captive Baboons kept in Knowsley Safari. Branches are coloured according to their cluster membership (A through I). *Strongyloides fuelleborni* type I (maroon star) identified here from captive baboons in Knowsley Safari (UK) is introduced for the first time. Coloured peripheral bars indicate the host species from which worms were isolated; dogs (Do), humans (Hu), chimpanzees (Ch), lorises (Lo), long-tailed macaques (Lt), pig-tailed macaques (Pt), Japanese macaques (Jm), proboscis monkeys (Pr), silvered leaf monkeys (Sl), orangutans (Or), Rhesus macaques (Rh), St. Kitts (white star) vervets (Ve), gorillas (Go) and baboons (Ba). Divergent *S. fuelleborni* types described by Ko et al*.* (2023) that have not been assigned a lineage are also shown (green circle and branches) from Siamang (Si), Douc (Do) and Francois’ langur (Fr) kept in zoological parks in Japan. The black bar and black star indicate *S. stercoralis* reference strain PV001. *Strongyloides stercoralis* types A and B are shown with red and blue branches, respectively. The loris clade is shown in light blue. Branches of the latter *S. stercoralis* and ‘loris’ clades are shaded grey to indicate non-fuelleborni *Strongyloides*. This same tree is provided in Supplementary File S3, though with isolate names shown on the branch tips.

**Figure 4.**
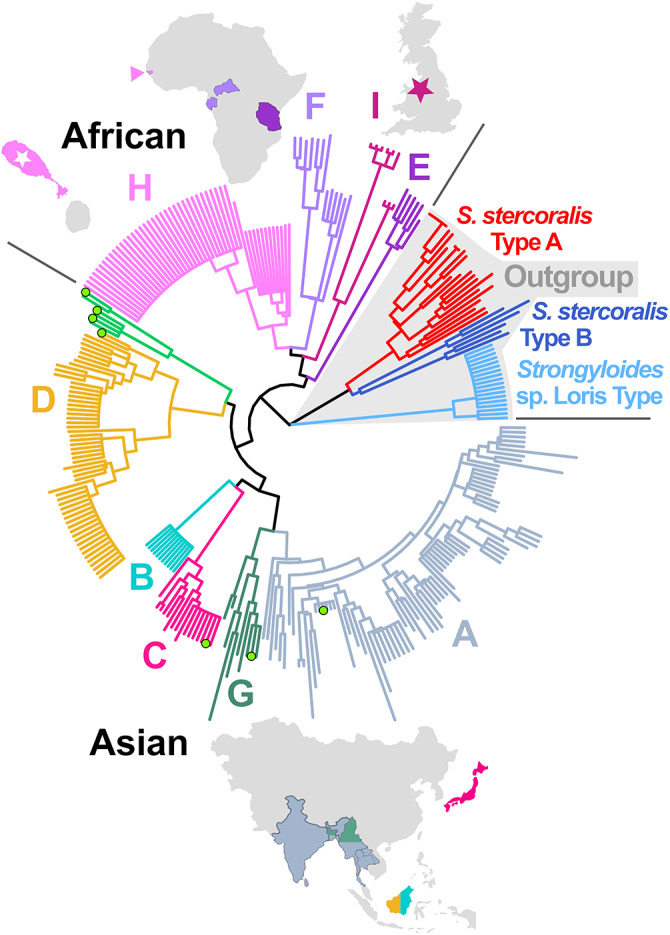
Neighbour-joining tree generated from reference *Strongyloides* genotypes and UK worms. This rooted tree was generated using the neighbour-joining method applied to a pairwise distance matrix computed from 309 *Strongyloides* genotypes constructed from available *cox1*, HVR-I and HVR-IV sequences, including the nine genotypes from *S. fuelleborni* isolated from captive Baboons kept in Knowsley Safari. Branches are coloured according to their cluster membership (A through I). Divergent *S. fuelleborni* types described by Ko *et al.* (2023) are also shown (bright green branches with no cluster/clade designation) from Siamang, Douc and Francois’ langur. African primates introduced to the island of St. Kitts are indicated in pink (with a white star) and those kept in Knowsley Safari are indicated in maroon (with a maroon star). Green circles on branch tips represent *cox1* sequences extracted from mt genome sequences of Asian *S. fuelleborni* types from Ko *et al.* (2023) that correspond to mt genomes used to construct the maximum likelihood phylogeny in Figure 5. This same tree though with isolate names shown on the branch tips is provided in Supplementary File S4.

**Figure 5.**
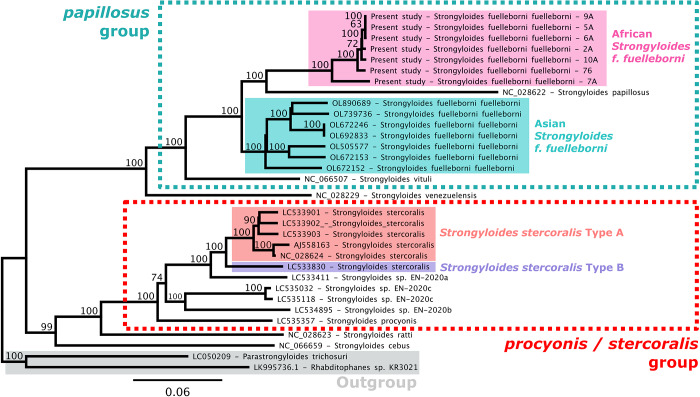
Evolutionary relationships among *Strongyloides* based on 14 mt genes. This tree represents a maximum-likelihood phylogeny of 14 mt genes from 30 *Strongyloides* species isolates plus single isolates of *Parastrongyloides trichosuri* and *Rabditophanes* sp. The14 gene sequences were concatenated and aligned using MUSCLE to a total of 12,135 positions. Genes were concatenated in the following order: 12S rRNA, 16S rRNA, *atp6*, *cox1, cox2, cox3, cytb, nd1, nd2, nd3, nd4, nd4L, nd5* and *nd6*. Genetic distances were calculated from this concatenated alignment using the dist.ml function in the ‘Phangorn’ R package and a neighbour-joining tree was generated using the NJ function. A maximum-likelihood tree was optimized using the pml and optim.pml functions, applying the GTR+G+I substitution model and NNI rearrangement model to produce a tree with a log-likelihood of −113863.8. The bootstrap.pml function was used to calculate non-parametric bootstrap values across 1000 samples. Bootstrap values above 50 are shown. The tree was rooted at the node shared by *Parastrongyloides trichosuri* and *Rabditophanes*. The phylogeny was rendered using the ggtree package (R) and annotations manually added using GIMP. Key features are highlighted, including the outgroup (solid grey box), *Strongyloides stercoralis* types A and B (solid red and purple boxes, respectively) and *Strongyloides fuelleborni* types from Asian (solid teal box) and African (solid pink box) primates. Sequences from African *S. fuelleborni* included in this figure were sequenced in the present study from UK worms.

Clustering of the nine complete UK genotypes alongside 300 *Strongyloides* reference genotypes resulted in the assignment of UK worms to the African *S. fuelleborni* clade, yet as a novel lineage not previously described. This lineage was designated as *S. fuelleborni* Type I (Figures 3 and 4). Complete mt genomes were assembled for seven of these nine UK worms; two could not be completely assembled due to insufficient coverage in some regions. The order of genes encoded in the mt genome of UK worms (arrangement C) differed from *S. fuelleborni* arrangements A and B described by Ko et al. (2023) from Asian *S. fuelleborni*. Briefly, the 12S and 16S rRNA genes, and 12 protein-coding genes in arrangement C were in the same order as arrangements A and B, although the order of the tRNA genes differed in UK worms, also noting that arrangement A possesses 22 tRNA genes while arrangements B and C include 23 tRNA genes. A graphical depiction of these arrangements can be found in Supplementary File S1, Tab D. Furthermore, UK worms possess expanded intergenic regions of unknown function that increase their mt genome size to approximately 24 kilobases (kb) compared to around 16 kb for Asian *S. fuelleborni*. A maximum likelihood phylogeny constructed from 14 coding regions (Figure 5) supported that Asian and African *S. fuelleborni* are distinct, and possibly paraphyletic, with Asian *S. fuelleborni* placed in a basal position to African *S. fuelleborni* and *S. papillosus* despite that the order of mt genes in African and Asian *S. fuelleborni* is similar relative to *S. papillosus* which has a comparatively disparate gene arrangement. This phylogeny also supports the observations of Ko et al. (2023) who highlight the close relationship between *S. fuelleborni* and *S. papillosus* from ruminants (Figure 5), which belong to a distinct clade separate from *S. stercoralis* and carnivore-infecting *Strongyloides* species.

## Discussion

In the present study, we took advantage of obtaining fresh material of *S. fuelleborni* infecting a semi-captive colony of UK baboons within Knowsley Safari. The cultures yielded a mixture of filariform larvae and free-living adult females with males rare to absent, consistent with past experimental studies on *S. fuelleborni* (Hansen et al., 1969). Various individual free-living adult females and filariform larvae were subjected to WGS; *cox1* and 18S rDNA genotypes extracted from these data confirmed a putative African ancestry for these worms and revealed that they belong to a novel lineage designated here as *S. fuelleborni* Type I. Complete mt genomes were assembled for seven UK worms revealing a unique mt gene arrangement, distinct from arrangements observed in Asian *S. fuelleborni* (Ko et al., 2023). These UK worms also possessed expanded mt intergenic regions of unknown function, making these mt genomes approximately 70% larger than those of Asian *S. fuelleborni*. This unusual molecular feature may have arisen from the unique populational founder and genetic drift effects of these worms as set within the peculiar mixed species baboon and drill ancestry of this semi-captive colony, which included various baboon species and a drill (Juhasz et al., 2023).

The maximum likelihood phylogeny we constructed from 14 mt coding regions supported a clear division between African and Asian *S. fuelleborni*, seemingly in contradiction to the long-held prevailing opinion that these populations represent a single species. In 1923, Hung and Hoeppli assigned the name *S. simiae* to isolates from Asian macaques, distinguishing it from African-origin *S. fuelleborni* on proposed morphologic grounds. However, the species-level distinction among populations of primate-infecting *Strongyloides* was largely rejected in the following decades, and the names were ultimately synonymized by Premvati (1959).

Despite this taxonomic revision based primarily on morphological similarity, other lines of evidence pre-dating genetic studies hinted at the legitimacy of the distinction between groups of primate-infecting *Strongyloides.* Early hybridization experiments by Augustine (1940) among *Pan* (African), *Macaca* (Asian) and *Cebus* (South American)-derived strains demonstrated that embryos that failed to develop in eggs produced by female worms following these crosses. This led Augustine (1940) to conclude that ‘each strain has satisfied the severest of all tests for deciding the validity of doubtful species, and accordingly we have: *Strongyloides fuelleborni* von Linstow, 1905, in *Pan troglodytes*; *S. simiae* Hung See Lu and Hoeppli, 1923, in *Macaca* sp. (probably *M. mulatta*)’. Considering this evidence along with a growing body of genetic data, the potential resurrection of *S. simiae* for Asian-origin *S. fuelleborni* becomes an intriguing prospect should further characterization continue to support its distinctiveness.

At a higher taxonomic level, the present work also suggests that a single clade occupied by *S. papillosus* and African *S. fuelleborni* represents the sister clade to Asian *S. fuelleborni*. However, only a single *S. paplillosis* mt genome has presently been made available in GenBank (NC_028622), limiting the present work to inclusion of only one set of *S. paplillosis* mt genes in the phylogeny. Analysis of additional *S. paplillosis* strains might impact the tree structure in such a way that *S. fuelleborni* becomes monophyletic. Similarly, Mt genomes from only a single variety of African *S. fuelleborni* (Type I) have been sequenced (to our knowledge), and analysis of additional varieties alongside those analysed here might also impact the tree structure. However, in light of the strong bootstrap support in the present analysis, the distinction between African and Asian *S. fuelleborni* seems well supported.

The results presented here support various hypotheses and underscore challenges faced by both historical and modern authors investigating *Strongyloides.* First, despite important differences in their host specialization, the apparently close but complicated relationship between primate *S. fuelleborni* and ruminant *S. papillosus* was demonstrated by our analysis. This relationship was proposed nearly a century ago based on morphologic and life cycle characteristics, although the status of unique species versus hostal varieties within this ‘egg laying, twisted ovaries’ group was a point of early debate (Chandler, 1925; Goodey, 1926). As discussed earlier, our observation that Asian and African *S. fuelleborni* clades were split by *S. papillosus* highlights the complexity of these relationships, which would be particularly challenging to investigate in the absence of genetic data. Clarification of this relationship could help resolve the still poorly understood evolutionary history of *S. fuelleborni.* The scenario of co-evolution and radiation with primates is compatible with its broad Old-World distribution and primate host diversity (Barratt and Sapp, 2020). On the other hand, it has also been proposed that the rarity of *S. fuelleborni* in ‘lower primates’ (prosimians) and its affinity with *S. papillosus* support a more recent host transfer/capture event (Ashford & Barnish, 1989). Further in-depth taxonomic study and isolate characterization are needed to fully understand this clade’s broader evolutionary history.

The variation in zoonotic potential and risk to humans among populations of *S. fuelleborni* and its relatives continues to be explored. Notably, it was originally assumed that only African strains of *S. fuelleborni* were capable of infecting humans (Ashford et al., 1992), while Asian *S. fuelleborni* was restricted to non-human primates and not important as a zoonotic parasite. Recently, however, several authors have discovered naturally acquired human infections with *S. fuelleborni* in Asia (Thanchomnang et al., 2017; Janwan et al., 2020; de Ree et al., 2024). Among Asian varieties of *S. fuelleborni*, Types A and G have been identified from humans. Among African *S. fuelleborni*, all types have been detected previously in humans, except for the new Type I described herein. Given the proven zoonotic characteristics of all other related African strains, it seems possible that Type I is capable of infecting humans as well. The presence of a putatively zoonotic strain in this captive population should be considered a potential risk for park visitors and staff and be mitigated accordingly.

One limitation of this work is that we still cannot address the phylogenetic position of the enigmatic subspecies *S. fuelleborni kelleyi*. Reports of *S. f. kellyi* are markedly absent from scientific literature published in recent decades and genetic data are scarce; only a 330 base pair fragment of the 18S rRNA gene attributed to *S. f. kelleyi* is available in GenBank (GB: AJ417029.1), and it is identical at the region captured to sequences attributed to *Strongyloides venezuelensis* (GB: LM525113.1, AB923887.1), *Strongyloides vituli* (GB: EU885229.1) and *Strongyloides cebus* (GB: AJ417025.1). Thus, given the lack of sequence information for *S. f. kellyi*, its phylogenetic relationship with *S. f. fuelleborni* and other *Strongyloides* species remains obscure (Barratt and Sapp, 2020).

To conclude, making good use of these UK worms, our work adds to the growing body of knowledge on the diversity of *S. fuelleborni* and contributes to our understanding of the phylogenetic relationship between various *Strongyloides* species. We were able to confirm that *S. fuelleborni* isolated from these UK baboons are of African ancestry and are uniquely distinct from Asian varieties of *S. fuelleborni*. The order of genes encoded within the mt genomes supports that Asian and African *S. fuelleborni* are more closely related to each other than to *S. papillosus*. However, the results of our phylogenetic analysis support that Asian and African *S. fuelleborni* populations may be paraphyletic, which could influence taxonomic designations of these populations if future investigations on biology, genetics and life cycle characteristics continue to support this distinction.

The genetic diversity of *S. stercoralis* and its influence on the epidemiology of human strongyloidiasis has been of substantial recent interest, while the human health significance of the ‘alternative’ *S. fuelleborni* strongyloidiasis is comparatively neglected. Developing tools for genotyping and identifying diagnostically informative markers rely on continual efforts to identify and characterize primate *Strongyloides* isolates globally. Genetic insights will continue to refine our understanding of the transmission patterns and public health risks posed by this zoonotic nematode.

## Supporting information

Richins et al. supplementary material Richins et al. supplementary material

## Supplementary material

The supplementary material for this article can be found at https://doi.org/10.1017/S0031182025100243.

## References

[ref1] Ashford R and Barnish G (1989) *Strongyloides fuelleborni*. In Grove DE (ed.), *Strongyloidiasis: A Major Roundworm Infection of Man*. Oxfordshire, UK: Taylor & Francis Group, 271–286.

[ref2] Ashford RW, Barnish G and Viney ME (1992) *Strongyloides fuelleborni* *kellyi*: Infection and disease in Papua New Guinea. *Parasitol Today* 8, 314–318. doi:10.1016/0169-4758(92)90106-c 15463651

[ref3] Augustine DL (1940) Experimental studies on the validity of species in the genus *Strongyloides*1. *American Journal of Epidemiology* 32-SectionD, 24–32. doi:10.1093/oxfordjournals.aje.a118677

[ref4] Barratt J, Houghton K, Richins T, Straily A, Threlkel R, Bera B, Kenneally J, Clemons B, Madison-Antenucci S, Cebelinski E, Whitney BM, Kreil KR, Cama V, Arrowood MJ and Qvarnstrom Y (2021) Investigation of US *Cyclospora cayetanensis* outbreaks in 2019 and evaluation of an improved *Cyclospora* genotyping system against 2019 cyclosporiasis outbreak clusters. *Epidemiology and Infec* 149, e214. doi:10.1017/S0950268821002090 PMC8506454 34511150

[ref5] Barratt JLN, Lane M, Talundzic E, Richins T, Robertson G, Formenti F, Pritt B, Verocai G, Nascimento de Souza J, Mato Soares N, Traub R, Buonfrate D and Bradbury RS (2019) A global genotyping survey of *Strongyloides stercoralis* and *Strongyloides fuelleborni* using deep amplicon sequencing. *PLoS Neglected Trop Dis* 13, e0007609. doi:10.1371/journal.pntd.0007609 PMC6762204 31525192

[ref6] Barratt JLN and Sapp SGH (2020) Machine learning-based analyses support the existence of species complexes for *Strongyloides fuelleborni* and *Strongyloides stercoralis*. *Parasitology* 147, 1184–1195. doi:10.1017/S0031182020000979 32539880 PMC7443747

[ref7] Buonfrate D, Hunt VL, Odermatt P and Streit A (2024) *Strongyloides*: Omics to worm-free populations. *Philosophical Transactions of the Royal Society of London B: Biological Sciences* 379, 20220448. doi:10.1098/rstb.2022.0448 38008116 PMC10676809

[ref8] Chandler AC (1925) The species of *Strongyloides* (Nematoda). *Parasitology* 17, 426–433. doi:10.1017/s0031182000004856

[ref9] Cunningham LJ, Nevin W, Verweij JJ, Buonfrate D, Scarso S, Khieu V, O’Ferrall AM, Rollason S and Stothard R (2025) Improving molecular epidemiological surveillance of strongyloidiasis upon differentiation of *Strongyloides fuelleborni fuelleborni* from *Strongyloides stercoralis*. *J Infect Dis* 27(jiaf237), 1–5. doi:10.1093/infdis/jiaf237. PMC12308652 40423557

[ref10] Dancescu P (1968) Investigations on the intensity of the infection in a strongyloidiasis focus. The coal culture method. *Trans Roy Soc Trop Med Hyg* 62, 490–495. doi:10.1016/0035-9203(68)90131-4 5677791

[ref11] de Ree V, Nath TC, Barua P, Harbecke D, Lee D, Rodelsperger C and Streit A (2024) Genomic analysis of *Strongyloides stercoralis* and *Strongyloides fuelleborni* in Bangladesh. *PLoS Neglected Trop Dis* 18, e0012440. doi:10.1371/journal.pntd.0012440 PMC11407627 39226300

[ref12] Frias L, Stark DJ, Lynn MS, Nathan SK, Goossens B, Okamoto M and MacIntosh AJJ (2018) Lurking in the dark: Cryptic *Strongyloides* in a Bornean slow loris. *Int J Parasitol Parasites Wildl* 7, 141–146. doi:10.1016/j.ijppaw.2018.03.003 29988792 PMC6031959

[ref13] Goodey T (1926) Observations on *Strongyloides fülleborni* von Linstow, 1905, with some remarks on the genus *Strongyloides*. *Journal of Helminthology* 4, 75–86. doi:10.1017/s0022149x00029576

[ref14] Hansen EL, Buecher EJ and Cryan WS (1969) *Strongyloides fulleborni*: Environmental factors and free-living generations. *Exp. Parasitol.* 26, 336–343. doi:10.1016/0014-4894(69)90127-1 5408381

[ref15] Hasegawa H, Sato H, Fujita S, Nguema PP, Nobusue K, Miyagi K, Kooriyama T, Takenoshita Y, Noda S, Sato A, Morimoto A, Ikeda Y and Nishida T (2010) Molecular identification of the causative agent of human strongyloidiasis acquired in Tanzania: Dispersal and diversity of *Strongyloides* spp. and their hosts. *Parasitology International* 59, 407–413. doi:10.1016/j.parint.2010.05.007 20621633

[ref16] Hung SL and Höppli R (1923) Morphologische und histologische beiträge zur *Strongyloides*-infection der tiere. *Arch. F. Schiffs-u. Tropen-Hyg* 27, 118–129.

[ref17] Hunt VL, Tsai IJ, Coghlan A, Reid AJ, Holroyd N, Foth BJ, Tracey A, Cotton JA, Stanley EJ, Beasley H, Bennett HM, Brooks K, Harsha B, Kajitani R, Kulkarni A, Harbecke D, Nagayasu E, Nichol S, Ogura Y, Quail MA, Randle N, Xia D, Brattig NW, Soblik H, Ribeiro DM, Sanchez-Flores A, Hayashi T, Itoh T, Denver DR, Grant W, Stoltzfus JD, Lok JB, Murayama H, Wastling J, Streit A, Kikuchi T, Viney M and Berriman M (2016) The genomic basis of parasitism in the *Strongyloides* clade of nematodes. *Nature Genetics [Internet]* 48, 299–307. doi:10.1038/ng.3495 26829753 PMC4948059

[ref18] Jacobson D, Zheng Y, Plucinski MM, Qvarnstrom Y and Barratt JLN (2022) Evaluation of various distance computation methods for construction of haplotype-based phylogenies from large MLST datasets. *Mol. Phylogenet. Evol* 177, 107608. doi:10.1016/j.ympev.2022.107608 35963590 PMC10127246

[ref19] Jaleta TG, Zhou S, Bemm FM, Schar F, Khieu V, Muth S, Odermatt P, Lok JB and Streit A (2017) Different but overlapping populations of *Strongyloides stercoralis* in dogs and humans-dogs as a possible source for zoonotic strongyloidiasis. *PLoS Neglected Trop Dis* 11, e0005752. doi:10.1371/journal.pntd.0005752 PMC5565190 28793306

[ref20] Janwan P, Rodpai R, Intapan PM, Sanpool O, Tourtip S, Maleewong W and Thanchomnang T (2020) Possible transmission of *Strongyloides fuelleborni* between working Southern pig-tailed macaques (*Macaca nemestrina*) and their owners in Southern Thailand: Molecular identification and diversity. *Infection Genetics & Evolution* 85, 104516. doi:10.1016/j.meegid.2020.104516 32860989

[ref21] Juhasz A, Spiers E, Tinsley E, Chapman E, Shaw W, Head M, Cunningham LJ, Archer J, Jones S, Haines LR, Davies Walsh N, Johnson B, Quayle J, Jones J, LaCourse EJ, Cracknell J and Stothard JR (2023) Gastrointestinal parasites in captive olive baboons in a UK safari park. *Parasitology* 150, 1096–1104. doi:10.1017/S0031182023000823 37655745 PMC10801365

[ref22] Ko PP, Haraguchi M, Hara T, Hieu DD, Ito A, Tanaka R, Tanaka M, Suzumura T, Ueda M, Yoshida A, Maruyama H and Nagayasu E (2023) Population genetics study of *Strongyloides fuelleborni* and phylogenetic considerations on primate-infecting species of *Strongyloides* based on their mitochondrial genome sequences. *Parasitology International* 92, 102663. doi:10.1016/j.parint.2022.102663 36058466

[ref23] Little M (1966) Comparative morphology of six species of *Strongyloides* (Nematoda) and redefinition of the genus. *The Journal of Parasitology* 52, 69–84. 5929983

[ref24] Lowe TM and Chan PP (2016) tRNAscan-SE On-line: Integrating search and context for analysis of transfer RNA genes. *Nucleic Acids Re* 44, W54–57. doi:10.1093/nar/gkw413 PMC4987944 27174935

[ref25] Nutman TB (2017) Human infection with *Strongyloides stercoralis* and other related *Strongyloides* species. *Parasitology* 144, 263–273. doi:10.1017/S0031182016000834 27181117 PMC5563389

[ref26] Premvati (1959) Studies on *Strongyloides* of primates: V. synonymy of the species in monkeys and apes. *Canadian Journal of Zoology* 37, 75–81.

[ref27] Richins T, Sapp SGH, Ketzis JK, Willingham AL, Mukaratirwa S, Qvarnstrom Y and Barratt JLN (2023) Genetic characterization of *Strongyloides fuelleborni* infecting free-roaming African vervets (*Chlorocebus aethiops sabaeus*) on the Caribbean island of St. Kitts. *Int J Parasitol Parasites Wildl* 20, 153–161. doi:10.1016/j.ijppaw.2023.02.003 36860205 PMC9969202

[ref28] Saitou N and Nei M (1987) The neighbor-joining method: A new method for reconstructing phylogenetic trees. *Mol. Biol. Evol.* 4, 406–425. doi:10.1093/oxfordjournals.molbev.a040454 3447015

[ref29] Sandground J (1925) Speciation and specificity in the nematode genus *Strongyloides*. *The Journal of Parasitology* 12, 59–80.

[ref30] Thanchomnang T, Intapan PM, Sanpool O, Rodpai R, Tourtip S, Yahom S, Kullawat J, Radomyos P, Thammasiri C and Maleewong W (2017) First molecular identification and genetic diversity of *Strongyloides stercoralis* and *Strongyloides fuelleborni* in human communities having contact with long-tailed macaques in Thailand. *Parasitology Research* 116, 1917–1923. doi:10.1007/s00436-017-5469-z 28500375

[ref31] Viney M, Ashford R and Barnish G (1991) A taxonomic study of *Strongyloides* Grassi, 1879 (Nematoda) with special reference to *Strongyloides fuelleborni* von Linstow, 1905 in man in Papua New Guinea and the description of a new subspecies. *Systematic Parasitology* 18, 95–109.

[ref32] von Linstow O (1905) *Strongyloides fulleborni*, n. sp. centralb bakteriol parasitenk infektionskrank. *Abt 1 Orig* 38, 532–533.

[ref33] Ward JH (1963) Hierarchical grouping to optimize an objective function. *Journal of the American Statistical Association* 58, 236–244. doi:10.1080/01621459.1963.10500845

[ref34] Yelifari L, Bloch P, Magnussen P, van Lieshout L, Dery G, Anemana S, Agongo E and Polderman AM (2005) Distribution of human *Oesophagostomum bifurcum*, hookworm and *Strongyloides stercoralis* infections in northern Ghana. *Trans Roy Soc Trop Med Hyg* 99, 32–38. doi:10.1016/j.trstmh.2004.02.007 15550259

