# Supplementary material for: Genetic diversity within Strongyloides fuelleborni: mitochondrial genome analysis reveals a clear African and Asian division
Source: Parasitology. 2025 Jun 23;152(7):735–44. doi: 10.1017/S0031182025100243 (PMC12418279; doi:10.1017/S0031182025100243)
Supplement: Richins et al. supplementary material [file S0031182025100243sup001.zip › S0031182025100243sup001/Supplementary File S2 - knowsley.docx]

**Supplemental File S2 for:**

Genetic diversity within *Strongyloides fuelleborni:* Mitochondrial genome analysis reveals a clear African and Asian division

Travis Richins^1^, Sarah G. H. Sapp^1^, Alexandra Juhasz^2^, Lucas J. Cunningham^2^, E. James LaCourse^2^, J. Russell Stothard^2^*, Joel L. N. Barratt^1^*

^1^Centers for Disease Control and Prevention, Division of Parasitic Diseases and Malaria, Laboratory Sciences and Diagnostic Branch, Atlanta, Georgia, USA

^2^ Department of Tropical Disease Biology, Liverpool School of Tropical Medicine, Pembroke Place, Liverpool, UK

*corresponding authors:

JLN Barratt -- ORCID ID of JLN Barratt: 0000-0001-8711-2408

**Email (J. L. N. Barratt):** [jbarratt@cdc.gov](mailto:jbarratt@cdc.gov)

JR Stothard -- ORCID ID of R Stothard: 0000-0002-9370-3420

**Email (R. Stothard):** [russell.stothard@lstmed.ac.uk](mailto:russell.stothard@lstmed.ac.uk)

# Table of contents

[Genetic characterization of *Strongyloides fuelleborni* infecting free-ranging captive baboons in a UK safari park i](#_Toc175299848)

[Table of contents ii](#_Toc175299849)

[Appendix A. Supplementary methods and other notes 1](#_Toc175299850)

[Defining cox1 segments 1](#_Toc175299851)

[Figure S1. Graphical depiction of cox1 amplicons generated in various studies relative to the 15-mer segments used in the present study for distance computation 1](#_Toc175299852)

[Notes on reference genotypes 2](#_Toc175299853)

[S. fuelleborni genotypes obtained from St Kitts vervets 2](#_Toc175299854)

[Mitochondrial reference genomes 2](#_Toc175299855)

[Appendix A. References 2](#_Toc175299856)

[Appendix B. Fasta sequences of each HVR-I haplotype 4](#_Toc175299857)

[Appendix C. Fasta sequences of each HVR-IV haplotype 6](#_Toc175299858)

[Appendix D. Fasta sequences of segmented haplotypes 8](#_Toc175299859)

#

# Appendix A. Supplementary methods and other notes

## Defining cox1 segments


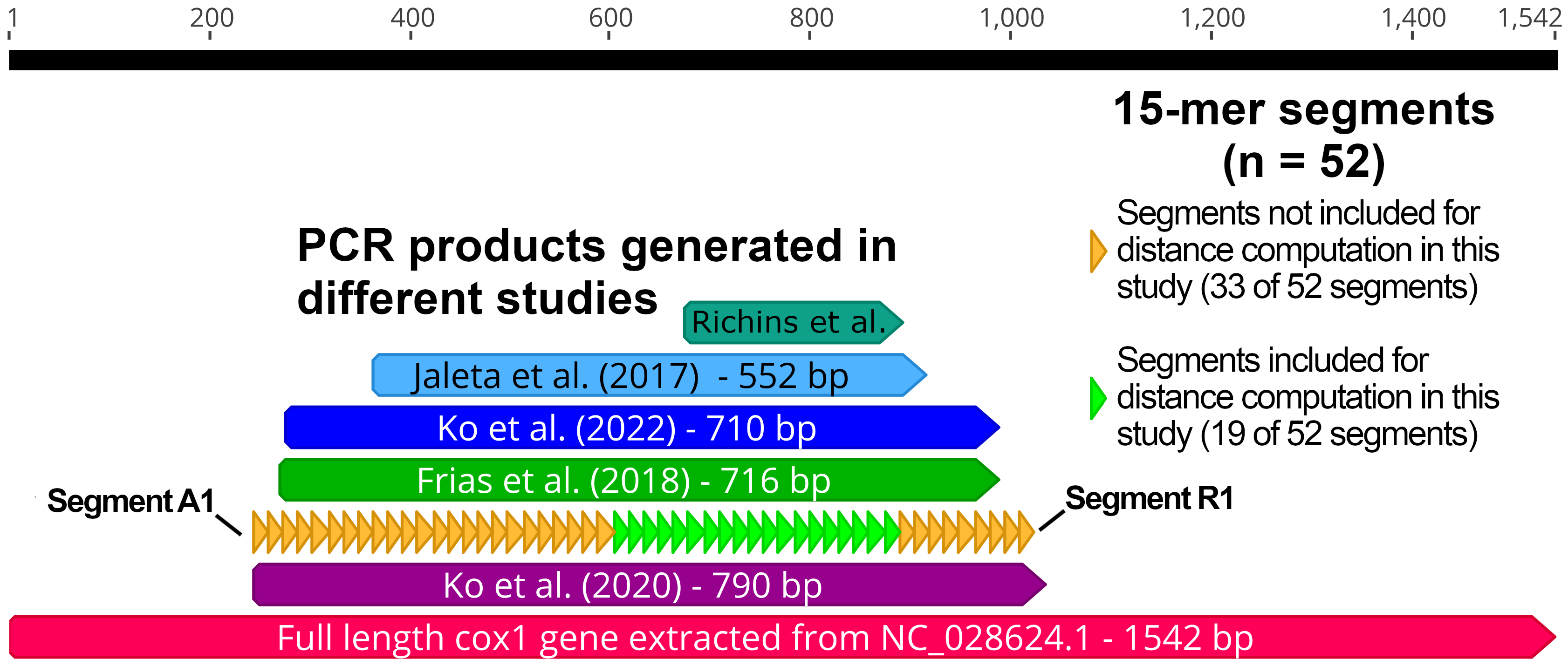


### Figure S1. Graphical depiction of cox1 amplicons generated in various studies relative to the 15-mer segments used in the present study for distance computation

Different regions of the *Strongyloides* cox1 gene and different combinations of HVR-I, HVR-IV and cox1 were sequenced in various independent *Strongyloides* genotyping studies. In the study by Richins et al. (2023), a cox1 region of 217 base pairs was sequenced. Ko et al. (2020) published amplicon sequences of cox1 that spanned the largest portion (790 base pairs) of the full-length cox1 gene. Barratts heuristic facilitates clustering of genotypes that vary in their degree of completeness (i.e., samples sequenced at different but overlapping combinations of loci may be included in the same analysis). This is because Barratt’s heuristic attempts to impute distances for loci that are available for some samples but missing for others. However, this requires that minimum data requirements be set by the investigator to ensure that an adequate amount of sequence data is available to provide the algorithm with a sufficient degree of initial context during these imputations. As per Richins et al. (2023) a phylogeny that reflects our current understanding of the *S. fuelleborni* population structure could be generated when using as few as 19 segments (specifically, the 285 bp section of cox1 indicated by green triangles above), plus various combinations of HVR-I and HVR-IV. In the present study, we extracted all 19 cox1 segments, plus HVR-I and HVR-IV sequences from the whole genome sequence data generated here from Knowsley Safari Park Strongylodies and utilized all of this data to cluster these samples. However, genotypes generated by Richins et al. (2023) from African vervets were only sequenced a 217 base pair amplicon of cox 1 (comprising 14 of the 19 segments highlighted above). Thus, Barratt’s heuristic would be required to impute distances for 5 segments for the samples from Richins et al. (2023) when computing genetic distances.

## Notes on reference genotypes

### S. fuelleborni genotypes obtained from St Kitts vervets

All *S. fuelleborni* genotypes utilized in this study are described in File S2 (Tab A). Notably, the *S. fuelleborni* genotypes described from free roaming African vervet monkeys were generated via amplification of cox1 and 18S amplicons directly from stool (Richins et al., 2023). Consequently, individual fecal samples sometimes yielded multiple cox1 and 18S haplotypes. Specifically, the samples gave rise to 18S haplotypes that were mixtures of HVR-I haplotypes XII, XVI and XVII (African-type *S. fuelleborni* haplotypes), mixtures of HVR-IV haplotypes L, T, M, O, R and P (also African-type *S. fuelleborni* haplotypes), and multiple cox1 haplotypes. Thus, it was not possible to construct a complete genotype as it could not be ascertained which particular cox1 haplotype might belong to a given HVR-I or HVR-IV haplotype in these mixed populations. To address this, each novel cox1 sequence that was detected in a sample was designated as an individual genotype. Next, to complement these cox1 sequences with additional 18S data (to improve distance computation accuracy), segments B and C of HVR-I were added to these genotypes as were segments A and C of HVR-IV. This is because HVR-I segments B and C are conserved/identical across all of HVR-I haplotypes XII, XVI and XVII. Similarly, segments A and C of HVR-IV are conserved across all of haplotypes L, T, M, O, R and P. Therefore, *S. fuelleborni* genotypes from vervets represented in the haplotype datasheet (File S2, Tab B) only contain these particular 18S segments.

## Mitochondrial genomes used to construct a maximum likelihood phylogeny

### Table S1. Reference genomes used for phylogeny

| **Species** | **GenBank Accession** | **Reference** | **Notes** |
| --- | --- | --- | --- |
| *Strongyloides vituli* | NC_066507 | - | Isolated from cow (*Bos taurus*) feces in Japan. |
| *Strongyloides venezuelensis* | NC_028229 | Hunt et al. (2016) | Isolate: HH1 |
| *Strongyloides stercoralis* | LC533903 | Ko et al. (2020) | Strain: P11_8. An A-type *S. stercoralis.* |
| *Strongyloides stercoralis* | LC533901 | Ko et al. (2020) | Strain: MYZ001_8. An A-type *S. stercoralis* |
| *Strongyloides stercoralis* | NC_028624 | Hunt et al. (2016) | Strain: PV001. An A-type *S. stercoralis* belonging to the VI-A group. |
| *Strongyloides stercoralis* | LC533902 | Ko et al. (2020) | Strain: HTB177_4. An A-type *S. stercoralis.* |
| *Strongyloides stercoralis* | LC533830 | Ko et al. (2020) | Strain: D024_2. A B-type *S. stercoralis.* |
| *Strongyloides stercoralis* | AJ558163 | Hu et al. (2003) | An A-type *S. stercoralis* belonging to the VI-A group. |
| *Strongyloides* sp. EN-2020c | LC535118 | Ko et al. (2020) | Isolate: MNB642_1. From a raccoon (*Procyon lotor*) in Japan. |
| *Strongyloides* sp. EN-2020c | LC535032 | Ko et al. (2020) | Isolate: KB5*.* From a Japanese badger (*Meles anakuma*). |
| *Strongyloides* sp. EN-2020b | LC534895 | Ko et al. (2020) | Isolate: 1906. From a Siberian Weasel (*Mustela sibirica*) in Japan. |
| *Strongyloides* sp. EN-2020a | LC533411 | Ko et al. (2020) | Isolate: Dec6_029_7. From a domestic cat (*Felis catus*) in Japan. |
| *Strongyloides ratti* | NC_028623 | Hunt et al. (2016) | Isolate: ED321. From wild rats. |
| *Strongyloides procyonis* | LC535357 | Ko et al. (2020) | Isolate: TNB2596_01. From a raccoon (*Procyon lotor*) in Japan. |
| *Strongyloides papillosus* | NC_028622 | Hunt et al. (2016) | Isolate: LIN. From naturally-infected lambs in Germany. |
| *Strongyloides fuelleborni fuelleborni* | OL890689 | Ko et al. (2023) | Isolate: UTAN001_2. From *Pongo pygmaeus* (captive Bornean orangutan). Asian-type. |
| *Strongyloides fuelleborni fuelleborni* | OL739736 | Ko et al. (2023) | Isolate: TENAGA001. From *Symphalangus syndactylus* (captive siamang) in Japan. Asian-type. |
| *Strongyloides fuelleborni fuelleborni* | OL692833 | Ko et al. (2023) | Isolate: RSDL001. From Red-shanked douc (*Pygathrix nemaeus*) in Japan. Asian-type. |
| *Strongyloides fuelleborni fuelleborni* | OL672153 | Ko et al. (2023) | Isolate: MM11_7. From *Macaca mulatta* (Rhesus macaque) in Myanmar. Asian-type. |
| *Strongyloides fuelleborni fuelleborni* | OL505577 | Ko et al. (2023) | Isolate: MM11_5. From *Macaca mulatta* (Rhesus macaque) in Myanmar. Asian-type. |
| *Strongyloides fuelleborni fuelleborni* | OL672152 | Ko et al. (2023) | Isolate: KJM002B_2. From Macaca fuscata fuscata (Japanese macaque) in Japan. Asian-type. |
| *Strongyloides fuelleborni fuelleborni* | OL672246 | Ko et al. (2023) | Isolate: FLM001_3. From *Trachypithecus francoisi* (captive Francois' Langur) in Japan. Asian-type. |
| *Strongyloides cebus* | NC_066659 | Ko et al. (2023) | Isolate: SIMIRI001_4. From Saimiri boliviensis (captive black-capped squirrel monkey) in Japan. |
| *Strongyloides fuelleborni fuelleborni* | PV564808 | Present study. | Isolate: 10A. From captive baboons in the United Kingdom. African-type. |
| *Strongyloides fuelleborni fuelleborni* | PV564806 | Present study. | Isolate: 7A. From captive baboons in the United Kingdom. African-type. |
| *Strongyloides fuelleborni fuelleborni* | PV564809 | Present study. | Isolate: 76. From captive baboons in the United Kingdom. African-type. |
| *Strongyloides fuelleborni fuelleborni* | PV564803 | Present study. | Isolate: 2A. From captive baboons in the United Kingdom. African-type. |
| *Strongyloides fuelleborni fuelleborni* | PV564804 | Present study. | Isolate: 5A. From captive baboons in the United Kingdom. African-type. |
| *Strongyloides fuelleborni fuelleborni* | PV564805 | Present study. | Isolate: 6A. From captive baboons in the United Kingdom. African-type. |
| *Strongyloides fuelleborni fuelleborni* | PV564807 | Present study. | Isolate: 9A. From captive baboons in the United Kingdom. African-type. |
| *Parastrongyloides trichosuri* | LC050209 | Hunt et al. (2016) | Isolate: KNP. Used as outgroup in phylogeny. |
| *Rhabditophanes* sp. KR3021 | LK995736 | Hunt et al. (2016) | Strain: KR3021. Used as outgroup in phylogeny. |

Note: Excluding the tRNA genes, coding regions of the mitochondrial genomes from each of the strains listed in Table S1 were extracted and concatenated in the following order: 12S rRNA, 16S rRNA, atp6, cox1, cox2, cox3, cytb, nd1, nd2, nd3, nd4, nd4L, nd5, and nd6. An alignment of these concatenated sequences is provided as a fasta file in Supplementary File S3.

# Candidate tRNA genes identified using tRNAscan

>2A.trna1-LeuTAA (3556-3612) Leu (TAA) 57 bp Sc: 15.4

GTTGGTGTAGTATAATATAATACATCTGCTTTAAGCGCAGAAGCTTTATCTCCAACT

>2A.trna2-LysTTT (8801-8863) Lys (TTT) 63 bp Sc: 16.7

GACTGGTTAGCTTAAGTATAAAGCACTAGACTTTTAATCTGGGGATGGCGGGACCACCGG

TTA

>2A.trna3-AspGTC (18200-18258) Asp (GTC) 59 bp Sc: 16.5

AAAGCTTTAGTGTAACTGTAGCATATTCCACTGTCAATGGAAAGGTAGTTAAGGCTTTA

>5A.trna1-LeuTAA (3555-3611) Leu (TAA) 57 bp Sc: 15.4

GTTGGTGTAGTATAATATAATACATCTGCTTTAAGCGCAGAAGCTTTATCTCCAACT

>5A.trna2-LysTTT (8752-8814) Lys (TTT) 63 bp Sc: 16.7

GACTGGTTAGCTTAAGTATAAAGCACTAGACTTTTAATCTGGGGATGGCGGGACCACCGG

TTA

>5A.trna3-AspGTC (18092-18150) Asp (GTC) 59 bp Sc: 16.5

AAAGCTTTAGTGTAACTGTAGCATATTCCACTGTCAATGGAAAGGTAGTTAAGGCTTTA

>6A.trna1-LeuTAA (3555-3611) Leu (TAA) 57 bp Sc: 15.4

GTTGGTGTAGTATAATATAATACATCTGCTTTAAGCGCAGAAGCTTTATCTCCAACT

>6A.trna2-LysTTT (8798-8860) Lys (TTT) 63 bp Sc: 16.7

GACTGGTTAGCTTAAGTATAAAGCACTAGACTTTTAATCTGGGGATGGCGGGACCACCGG

TTA

>6A.trna3-AspGTC (18195-18253) Asp (GTC) 59 bp Sc: 16.5

AAAGCTTTAGTGTAACTGTAGCATATTCCACTGTCAATGGAAAGGTAGTTAAGGCTTTA

>7A.trna1-LeuTAA (4136-4192) Leu (TAA) 57 bp Sc: 15.4

GTTGGTGTAGTATAATATAATACATCTGCTTTAAGCGCAGAAGCTTTATCTCCAACT

>7A.trna2-LysTTT (8862-8924) Lys (TTT) 63 bp Sc: 16.7

GACTGGTTAGCTTAAGTATAAAGCACTAGACTTTTAATCTGGGGATGGCGGGACCACCGG

TTA

>7A.trna3-AspGTC (17840-17898) Asp (GTC) 59 bp Sc: 16.9

AAAGCTTTAGTGTAACTGTAGCATATCCCACTGTCAATGGGAAGGTAGTTAAGGCTTTA

>9A.trna1-LeuTAA (3555-3611) Leu (TAA) 57 bp Sc: 15.4

GTTGGTGTAGTATAATATAATACATCTGCTTTAAGCGCAGAAGCTTTATCTCCAACT

>9A.trna2-LysTTT (8752-8814) Lys (TTT) 63 bp Sc: 16.7

GACTGGTTAGCTTAAGTATAAAGCACTAGACTTTTAATCTGGGGATGGCGGGACCACCGG

TTA

>9A.trna3-AspGTC (18092-18150) Asp (GTC) 59 bp Sc: 16.5

AAAGCTTTAGTGTAACTGTAGCATATTCCACTGTCAATGGAAAGGTAGTTAAGGCTTTA

>10A.trna1-LeuTAA (3534-3590) Leu (TAA) 57 bp Sc: 15.4

GTTGGTGTAGTATAATATAATACATCTGCTTTAAGCGCAGAAGCTTTATCTCCAACT

>10A.trna2-LysTTT (8776-8838) Lys (TTT) 63 bp Sc: 16.7

GACTGGTTAGCTTAAGTATAAAGCACTAGACTTTTAATCTGGGGATGGCGGGACCACCGG

TTA

>10A.trna3-AspGTC (18176-18234) Asp (GTC) 59 bp Sc: 16.5

AAAGCTTTAGTGTAACTGTAGCATATTCCACTGTCAATGGAAAGGTAGTTAAGGCTTTA

>WORM_76.trna1-LeuTAA (3857-3913) Leu (TAA) 57 bp Sc: 15.4

GTTGGTGTAGTATAATATAATACATCTGCTTTAAGCGCAGAAGCTTTATCTCCAACT

>WORM_76.trna2-LysTTT (9095-9157) Lys (TTT) 63 bp Sc: 16.7

GACTGGTTAGCTTAAGTATAAAGCACTAGACTTTTAATCTGGGGATGGCGGGACCACCGG

TTA

# Appendix A. References

HU, M., CHILTON, N. B. & GASSER, R. B. 2003. The mitochondrial genome of Strongyloides stercoralis (Nematoda) - idiosyncratic gene order and evolutionary implications. *Int J Parasitol,* 33**,** 1393-408.

HUNT, V. L., TSAI, I. J., COGHLAN, A., REID, A. J., HOLROYD, N., FOTH, B. J., TRACEY, A., COTTON, J. A., STANLEY, E. J., BEASLEY, H., BENNETT, H. M., BROOKS, K., HARSHA, B., KAJITANI, R., KULKARNI, A., HARBECKE, D., NAGAYASU, E., NICHOL, S., OGURA, Y., QUAIL, M. A., RANDLE, N., XIA, D., BRATTIG, N. W., SOBLIK, H., RIBEIRO, D. M., SANCHEZ-FLORES, A., HAYASHI, T., ITOH, T., DENVER, D. R., GRANT, W., STOLTZFUS, J. D., LOK, J. B., MURAYAMA, H., WASTLING, J., STREIT, A., KIKUCHI, T., VINEY, M. & BERRIMAN, M. 2016. The genomic basis of parasitism in the Strongyloides clade of nematodes. *Nat Genet,* 48**,** 299-307.

KO, P. P., HARAGUCHI, M., HARA, T., HIEU, D. D., ITO, A., TANAKA, R., TANAKA, M., SUZUMURA, T., UEDA, M., YOSHIDA, A., MARUYAMA, H. & NAGAYASU, E. 2023. Population genetics study of *Strongyloides fuelleborni* and phylogenetic considerations on primate-infecting species of *Strongyloides* based on their mitochondrial genome sequences. *Parasitol Int,* 92**,** 102663.

KO, P. P., SUZUKI, K., CANALES-RAMOS, M., AUNG, M., HTIKE, W. W., YOSHIDA, A., MONTES, M., MORISHITA, K., GOTUZZO, E., MARUYAMA, H. & NAGAYASU, E. 2020. Phylogenetic relationships of *Strongyloides* species in carnivore hosts. *Parasitol Int,* 78**,** 102151.

RICHINS, T., SAPP, S. G. H., KETZIS, J. K., WILLINGHAM, A. L., MUKARATIRWA, S., QVARNSTROM, Y. & BARRATT, J. L. N. 2023. Genetic characterization of Strongyloides fuelleborni infecting free-roaming African vervets (Chlorocebus aethiops sabaeus) on the Caribbean island of St. Kitts. *Int J Parasitol Parasites Wildl,* 20**,** 153-161.

# Appendix B. Fasta sequences of each HVR-I haplotype

>Haplotype_I

AATATTTTAGTTGGATAACTGAGGTAATTCTTGAGCTAATACACGCTATTTATACCACATTAGTGGTGCGTTTATTTGATTAAACCATTTTATATTGGTTGACTCAAAATATCCTCGCTGATTTTGTTACTAAAACATACCGTATGTGTATCTGGTTTATCAACTTTCGATGGTAGGGTATTGGCCTACCATGGTTGTGACGGATAACGGAGAATTAGGGTTCGACTCCGGAGAGGGAGCCTGAGAAACGGCTACCACATCCAAGGAAGGCAGCAGGCGCGAAAATTACCCAATTTTAGTTAAAAGAGGTAGTGACGAAAAATGACAACCAAATATTATTATTAATATTTGGATTGAAAATCTTCAAGTTTAAATAACTTGTTGGTAAAGGAAAGGGCAAGTCTGGTGCCAGCAGCCGCGGTAATACCAGCTTT

>Haplotype_II

AATATTTTAGTTGGATAACTGAGGTAATTCTTGAGCTAATACACGCTATTTATACCACATTAGTGGTGCGTTTATTTGATTAAACCATTTTTATATTGGTTGACTCAAAATATCCTCGCTGATTTTGTTACTAAAACATACCGTATGTGTATCTGGTTTATCAACTTTCGATGGTAGGGTATTGGCCTACCATGGTTGTGACGGATAACGGAGAATTAGGGTTCGACTCCGGAGAGGGAGCCTGAGAAACGGCTACCACATCCAAGGAAGGCAGCAGGCGCGAAAATTACCCAATTTTAGTTAAAAGAGGTAGTGACGAAAAATGACAACCAAATATTATTATTAATATTTGGATTGAAAATCTTCAAGTTTAAATAACTTGTTGGTAAAGGAAAGGGCAAGTCTGGTGCCAGCAGCCGCGGTAATACCAGCTTT

>Haplotype_III

AATATTTTAGTTGGATAACTGAGGTAATTCTTGAGCTAATACACGCTATTTATACCACATTAGTGGTGCGTTTATTTGATTAAACCATTTTTATATTGGTTGACTCAAAATATCCTCGCTGATTTTGTTACTAAAACATACCGTATGTGTATCTGGTTTATCAACTTTCGATGGTAGGGTATTGGCCTACCATGGTTGTGACGGATAACGGAGAATTAGGGTTCGACTCCGGAGAGGGAGCCTGAGAAACGGCTACCACATCCAAGGAAGGCAGCAGGCGCGAAAATTACCCAATTTTAGTTAAAAGAGGTAGTGACGAAAAATGACAACCAAATATTATTATTAATATTTGGATTGAAAATCTTCAAGTATAAATAACTTGTTGGTAAAGGAAAGGGCAAGTCTGGTGCCAGCAGCCGCGGTAATACCAGCTTT

>Haplotype_IV

AATATTTTAGTTGGATAACTGAGGTAATTCTTGAGCTAATACACGCTATTTATACCACATTAGTGGTGCGTTTATTTGATTAAACCATTTATACTGGTTGACTCAAAATATCCTCGCTGATTTTGTTACTAAAACATACCGTATGTGTATCTGGTTTATCAACTTTCGATGGTAGGGTATTGGCCTACCATGGTTGTGACGGATAACGGAGAATTAGGGTTCGACTCCGGAGAGGGAGCCTGAGAAACGGCTACCACATCCAAGGAAGGCAGCAGGCGCGAAAATTACCCAATTTTAGTTAAAAGAGGTAGTGACGAAAAATGACAACCAAATATTATTATTAATATTTGGATTGAAAATCTTCAAGTTTAAATAACTTGTTGGTAAAGGAAAGGGCAAGTCTGGTGCCAGCAGCCGCGGTAATACCAGCTTT

>Haplotype_V

AATATTTTAGTTGGATAACTGAGGTAATTCTTGAGCTAATACACGCTATTTATACCACATTAGTGGTGCGTTTATTTGATTAAACCATTTTATACTGGTTGACTCAAAATATCCTCGCTGATTTTGTTACTAAAACATACCGTATGTGTATCTGGTTTATCAACTTTCGATGGTAGGGTATTGGCCTACCATGGTTGTGACGGATAACGGAGAATTAGGGTTCGACTCCGGAGAGGGAGCCTGAGAAACGGCTACCACATCCAAGGAAGGCAGCAGGCGCGAAAATTACCCAATTTTAGTTAAAAGAGGTAGTGACGAAAAATGACAACCAAATATTATTATTAATATTTGGATTGAAAATCTTCAAGTTTAAATAACTTGTTGGTAAAGGAAAGGGCAAGTCTGGTGCCAGCAGCCGCGGTAATACCAGCTTT

>Haplotype_VI

AATATTTTAGTTGGATAACTGAGGTAATTCTTGAGCTAATACACGCTATTTATACCACATTAGTGGTGCGTTTATTTGATTAAACCATTTTATATTGGTTGACTCAAAATATCCTCGCTGATTTTGTTACTAAAACATACCGTATGTGTATCTGGTTTATCAACTTTCGATGGTAGGGTATTGGCCTACCATGGTTGTGACGGATAACGGAGAATTAGGGTTCGACTCCGGAGAGGGAGCCTGAGAAACGGCTACCACATCCAAGGAAGGCAGCAGGCGCGAAAATTACCCAATTTTAGTTAAAAGAGGTAGTGACGAAAAATGACAACCAAATATTATTATTAATATTTGGATTGAAAATCTTCAAGTATAAATAACTTGTTGGTAAAGGAAAGGGCAAGTCTGGTGCCAGCAGCCGCGGTAATACCAGCTTT

>Haplotype_VII

AATATTTTAGTTGGATAACTGAGGTAATTCTTGAGCTAATACACGCTATTTATACCACATTAGTGGTGCGTTTATTTGATTAAACCATTTATATTGGTTGACTCAAAATATCCTCGCTGATTTTGTTACTAAAACATACCGTATGTGTATCTGGTTTATCAACTTTCGATGGTAGGGTATTGGCCTACCATGGTTGTGACGGATAACGGAGAATTAGGGTTCGACTCCGGAGAGGGAGCCTGAGAAACGGCTACCACATCCAAGGAAGGCAGCAGGCGCGAAAATTACCCAATTTTAGTTAAAAGAGGTAGTGACGAAAAATGACAACCAAATATTATTATTAATATTTGGATTGAAAATCTTCAAGTTTAAATAACTTGTTGGTAAAGGAAAGGGCAAGTCTGGTGCCAGCAGCCGCGGTAATACCAGCTTT

>Haplotype_VIII

AATATTTTAGTTGGATAACTGAGGTAATTCTTGAGCTAATACACGCTGTTAATACCACATTAGTGGTGCGTTTATTTGATTAAACCATTATATTGGTTGACTCAAAATATCCTCGCTGATTTTGTTACTAAAACATACCGTATGTGTATCTGGTTTATCAACTTTCGATGGTAGGGTATTGGCCTACCATGGTTGTGACGGATAACGGAGAATTAGGGTTCGACTCCGGAGAGGGAGCCTGAGAAACGGCTACCACATCCAAGGAAGGCAGCAGGCGCGAAAATTACCCAATTTTAGTTAAAAGAGGTAGTGACGAAAAATGACAACCAAATATTATTATTAATATTTGGATTGAAAATCTTCAAGTTTAAATAACTTGTTGGTAAAGGAAAGGGCAAGTCTGGTGCCAGCAGCCGCGGTAATACCAGCTTT

>Haplotype_IX

AATATTTTAGTTGGATAACTGAGGTAATTCTTGAGCTAATACACGCTACCTATACCACATTAGTGGTGCGTTTATTTGATTAAACCATTATTTTGGTTGACTCAAAATATCCTCGCTGATTTTGTTACTAAAACATACCGTATGTGTATCTGGTTTATCAACTTTCGATGGTAGGGTATTGGCCTACCATGGTTGTGACGGATAACGGAGAATTAGGGTTCGACTCCGGAGAGGGAGCCTGAGAAACGGCTACCACATCCAAGGAAGGCAGCAGGCGCGAAAATTACCCAATTTTAGTTTAAAGAGGTAGTGACGAAAAATGACAACCAAATATTATTATTAATATTTGGATTGAAAATCTTCAAGTTTAAATAACTTGTTGGTAAAGGAAAGGGCAAGTCTGGTGCCAGCAGCCGCGGTAATACCAGCTTT

>Haplotype_X

ACTATTTTAGTTGGATAACTGAGGTAATTCTTGAGCTAATACACGCTATTTATACCACATTAGTGGTGCGTTTATTTGATTAAACCATTATTTTGGTTGACTCAAAATATCCTCGCTGATTTTGTTACAAAAACATACCGTATGTGTATCTGGTTTATCAACTTTCGATGGTAGGGTATTGGCCTACCATGGTTGTGACGGATAACGGAGAATTAGGGTTCGACTCCGGAGAGGGAGCCTGAGAAACGGCTACCACATCCAAGGAAGGCAGCAGGCGCGAAAATTACCCAATTTTAGTTTAAAGAGGTAGTGACGAAAAATGACAACCAAATATTATTATTAATATTTGGATTGAAAATCTTCAAGTTTAAATAACTTGTTGGTAAAGGAAAGGGCAAGTCTGGTGCCAGCAGCCGCGGTAATACCAGCTTT

>Haplotype_XI

AATATTTTAGTTGGATAACTGAGGTAATTCTTGAGCTAATACACGCTATTTATACCACATTAGTGGTGCGTTTATTTGATTAAACCATTTTTATATATTGGTTGACTCAAAATATCCTCGCTGATTTTGTTACTAAAACATACCGTATGTGTATCTGGTTTATCAACTTTCGATGGTAGGGTATTGGCCTACCATGGTTGTGACGGATAACGGAGAATTAGGGTTCGACTCCGGAGAGGGAGCCTGAGAAACGGCTACCACATCCAAGGAAGGCAGCAGGCGCGAAAATTACCCAATTTTAGTTAAAAGAGGTAGTGACGAAAAATGACAACCAAATATTATTATTAATATTTGGATTGAAAATCTTCAAGTTTAAATAACTTGTTGGTAAAGGAAAGGGCAAGTCTGGTGCCAGCAGCCGCGGTAATACCAGCTTT

>Haplotype_XII

AATATTTTAGTTGGATAACTGAGGTAATTCTTGAGCTAATACACGCTATTTATACCACATTAGTGGTGCGTTTATTTGATTAAACCATTATAACGGTTGACTCAAAATATCCTTGCTGATTTTGTTACTAAAACATACCGTATGTGTATCTGGTTTATCAACTTTCGATGGTAGGGTATTGGCCTACCATGGTTGTGACGGATAACGGAGAATTAGGGTTCGACTCCGGAGAGGGAGCCTGAGAAACGGCTACCACATCCAAGGAAGGCAGCAGGCGCGAAAATTACCCAATTTTAGTTCAAAGAGGTAGTGACGAAAAATGACAACCAAATATTATTATTAATATTTGGATTGAAAATCTTCAAGTTTAAATAACTTGTTGGTAAAGGAAAGGGCAAGTCTGGTGCCAGCAGCCGCGGTAATACCAGCTTT

>Haplotype_XIII

GTTGGATAACTGACGTAATTCTTGAGCTAATACACGCTATTTATACCACATTAGTGGTGCGTTTATTTGATTAAACCATTATAACGGTTGACTCAAAATATCCTTGCTGATTTTGTTACTAAAACATACCGTATGTGTATCTGGTTTATCAACTTTCGATGGTAGGGTATTGGCCTACCATGGTTGTGACGGATAACGGAGAATTAGGGTTCGACTCCGGAGAGGGAGCCTGAGAAACGGCTACCACATCCAAGGAAGGCAGCAGGCGCGAAAATTACCCAATTTTAGTTCAAAGAGGTAGTGACGAAAAATGACAACCAAATATTATTATTAATATTTGGATTGAAAATCTTCAAGTTTAAATAACTTGTTGGTAAAGGTTTGGGCAAGTCTGGTGCCAGCAGCCGCGGTAATACCAGCTTT

>Haplotype_XIV

AATATTTTAGTTGGATAACTGAGGTAATTCTTGAGCTAATACACGCTATTTATACCACATTAGTGGTGCGTTTATTTGATTAAACCATTTTAACGGTTGACTCAAAATATCCTTGCTGATTTTGTTACTAAAACATACCGTATGTGTATCTGGTTTATCAACTTTCGATGGTAGGGTATTGGCCTACCATGGTTGTGACGGATAACGGAGAATTAGGGTTCGACTCCGGAGAGGGAGCCTGAGAAACGGCTACCACATCCAAGGAAGGCAGCAGGCGCGAAAATTACCCAATTTTAGTTCAAAGAGGTAGTGACGAAAAATGACAACCAAATATTATTATTAATATTTGGATTGAAAATCTTCAAGTTTAAATAACTTGTTGGTAAAGGAAAGGGCAAGTCTGGTGCCAGCAGCCGCGGTAATACCAGCTTT

>Haplotype_XV

AATATTTTAGTTGGATAACTGAGGTAATTCTTGAGCTAATACACGCTATTTATACCACATTAGTGGTGCGTTTATTTGATTAAACCATTTTTATATTGGTTGACTCAAAATATCCTCGCTGATTTTGTTACTAAAACATACCGTATGTGTATCTGGTTTATCAACTTTCGATGGTAGGGTATTGGCCTACCATGGTTGTGACGGATAACGGAGAATTAGGGTTCGACTCCGGAGAGGGAGCCTGAGAAATGGCTACCACATCCAAGGAAGGCAGCAGGCGCGAAAATTACCCAATTTTAGTTAAAAGAGGTAGTGACGAAAAATGACAACCAAATATTATTATTAATATTTGGATTGAAAATCTTCAAGTATAAATAACTTGTTGGTAAAGGAAAGGGCAAGTCTGGTGCCAGCAGCCGCGGTAATACCAGCTTT

>Haplotype_XVI

AATATTTTAGTTGGATAACTGAGGTAATTCTTGAGCTAATACACGCTATTTATACCACATTAGTGGTGCGTTTATTTGATTAAACCATTATAACGGTTGACTCAAAATATCCTTGCTGATTTTGTTACTAAAACATACCGTATGTGTATCTGGTTTATCAACTTTCGATGGTAGGGTATTGGCCTACCATGGTTGTGACGGATAACGGAGAATTAGGGTTCGACTCCGGAGAGGGAGCCTGAGAAACGGCTACCACATCCAAGGAAGGCAGCAGGCGCGAAAATTACCCAATTTTAGTTCAAAGAGGTAGTGACGAAAAATGACAACCAAATATTATTATATTAATATTTGGATTGAAAATCTTCAAGTTTAAATAACTTGTTGGTAAAGGAAAGGGCAAGTCTGGTGCCAGCAGCCGCGGTAATACCAGCTTT

>Haplotype_XVII

AATATTTTAGTTGGATAACTGAGGTAATTCTTGAGCTAATACACGCTATTTATACCACATTAGTGGTGCGTTTATTTGATTAAACCATTATATAACGGTTGACTCAAAATATCCTTGCTGATTTTGTTACTAAAACATACCGTATGTGTATCTGGTTTATCAACTTTCGATGGTAGGGTATTGGCCTACCATGGTTGTGACGGATAACGGAGAATTAGGGTTCGACTCCGGAGAGGGAGCCTGAGAAACGGCTACCACATCCAAGGAAGGCAGCAGGCGCGAAAATTACCCAATTTTAGTTCAAAGAGGTAGTGACGAAAAATGACAACCAAATATTATTATTAATATTTGGATTGAAAATCTTCAAGTTTAAATAACTTGTTGGTAAAGGAAAGGGCAAGTCTGGTGCCAGCAGCCGCGGTAATACCAGCTTT

# Appendix C. Fasta sequences of each HVR-IV haplotype

>Haplotype_A

ATTGACAGATTGATAGCTCTTTCATGATTTAGTGGTTGGTGGTGCATGGCCGTTCTTAGTTCGTGGATATGATTTGTCTGGTTGATTCCGATAACGAGCGAGACTTTTATGTTATATTAAATATTATTATTTTGTTTATTTTAATATAAATAATTAATATTTTAATAACAGATTAATAGTGTTTAACTATTTGAGAGAGAGCGATAACAGGTCTGTGATGCCCTTAGATGTCCGGGGCTGCACGCGCGCTACAAT

>Haplotype_B

ATTGACAGATTGATAGCTCTTTCATGATTTAGTGGTTGGTGGTGCATGGCCGTTCTTAGTTCGTGGATATGATTTGTCTGGTTGATTCCGATAACGAGCGAGACTTTTATGTTATATTAAATATTATTATTTGTTTATTTTTTATATAAATAATTAATATTTTAATAACAGATTAATAGTGTTTAACTATTTGAGAGAGAGCGATAACAGGTCTGTGATGCCCTTAGATGTCCGGGGCTGCACGCGCGCTACAAT

>Haplotype_C

ATTGACAGATTGATAGCTCTTTCATGATTTAGTGGTTGGTGGTGCATGGCCGTTCTTAGTTCGTGGATATGATTTGTCTGGTTGATTCCGATAACGAGCGAGACTTTTATGTTATATTAAATATAATTATTTTGTTTATTTTAATATAAATAATTAATATTTTAATAACAGATTAATAGTGTTTAACTATTTGAGAGAGAGCGATAACAGGTCTGTGATGCCCTTAGATGTCCGGGGCTGCACGCGCGCTACAAT

>Haplotype_D

ATTGACAGATTGATAGCTCTTTCATGATTTAGTGGTTGGTGGTGCATGGCCGTTCTTAGTTCGTGGATATGATTTGTCTGGTTGATTCCGATAACGAGCGAGACTTTTATGTTATATTAAATATTATTATTTGTTTATTTTATATAAATAATTAATATTTTAATAACAGATTAATAGTGTTTAACTATTTGAGAGAGAGCGATAACAGGTCTGTGATGCCCTTAGATGTCCGGGGCTGCACGCGCGCTACAAT

>Haplotype_E

ATTGACAGATTGATAGCTCTTTCATGATTTAGTGGTTGGTGGTGCATGGCCGTTCTTAGTTCGTGGATATGATTTGTCTGGTTGATTCCGATAACGAGCGAGACTTTTATGTTATATTAAATATTATTATTTGTTTATTTTAATATAAATAATTAATATTTTAATAACAGATTAATAGTGTTTAACTATTTGAGAGAGAGCGATAACAGGTCTGTGATGCCCTTAGATGTCCGGGGCTGCACGCGCGCTACAAT

>Haplotype_F

ATTGACAGATTGATAGCTCTTTCATGATTTAGTGGTTGGTGGTGCATGGCCGTTCTTAGTTCGTGGATATGATTTGTCTGGTTGATTCCGATAACGAGCGAGACTTTTATGTTATATTAAATATTATTATTTTATTATTTTATATAAATAATTAATATTTTAATAACAGATTAATAGTGTTTAACTATTTGAGAGAGAGCGATAACAGGTCTGTGATGCCCTTAGATGTCCGGGGCTGCACGCGCGCTACAAT

>Haplotype_G

ATTGACAGATTGATAGCTCTTTCATGATCTAGTGGTTGGTGGTGCATGGCCGTTCTTAGTTCGTGGATATGATTTGTCTGGTTGATTCCGATAACGAGCGAGACTTTTATGTTATATTAAATATTATTATTTTGTTTATTTTAATATAAATAATTAATATTTTAATAACAGATTAATAGTGTTTAACTATTTGAGAGAGAGCGATAACGGGTCTGTGATGCCCTTAGATGTCCGGGGCTGCACGCGCGCTACAAT

>Haplotype_H

ATTGACAGATTGATAGCTCTTTCATGATTTAGTGGTTGGTGGTGCATGGCCGTTCTTAGTTCGTGGATATGATTTGTCTGGTTGATTCCGATAACGAGCGAGACTTTTATGTTATATTAAATATTATTATTTTATTATATAAATAATAATTATTTTAATAACAGATTAATAGTGTTTAACTATTTGAGAGAGAGCAATAACAGGTCTGTGATGCCCTTAGATGTCCGGGGCTGCACGCGCGCTACAAT

>Haplotype_I

ATTGACAGATTGATAGCTCTTTCATGATTTAGTGGTTGGTGGTGCATGGCCGTTCTTAGTTCGTGGATATGATTTGTCTGGTTGATTCCGATAACGAGCGAGACTTTTATGTTATATTAAATATTATTATTTTTTATATAAATAATAATTATTTTAATAACAGATTAATAGTGTTTAACTATTTGAGAGAGAGCAATAACAGGTCTGTGATGCCCTTAGATGTCCGGGGCTGCACGCGCGCTACAAT

>Haplotype_J

ATTGACAGATTGATAGCTCTTTCATGATTTAGTGGTTGGTGGTGCATGGCCGTTCTTAGTTCGTGGATATGATTTGTCTGGTTGATTCCGATAACGAGCGAGACTTTTATGTTATATTAAATATTATTATTTTGTTTATTTTAATATAAATAATTAATATTTTAATAACAGATTAATAGTGTTTAACTATTTGAGAGAGAGCGATAACAGGTATGTGATGCCCTTAGATGTCCGGGGCTGCACGCGCGCTACAAT

>Haplotype_K

ATTGACAGATTGATAGCTCTTTCATGATTTAGTGGTTGGTGGTGCATGGCCGTTCTTAGTTCGTGGATATGATTTGTCTGGTTGATTCCGATAACGAGCGAGACTTTTATGTTATATTAAATAATATTATTTATAAATTTTATATTTTATATAATATTTTATTTTAATAACAGATTAATAGTGTTTAACTATTTGAGAGAGAGCAATAACAGGTCTGTGATGCCCTTAGATGTCCGGGGCTGCACGCGCGCTACAAT

>Haplotype_L

ATTGACAGATTGATAGCTCTTTCATGATTTAGTGGTTGGTGGTGCATGGCCGTTCTTAGTTCGTGGATATGATTTGTCTGGTTGATTCCGATAACGAGCGAGACTTTTATGTTATATTAAATAATATTATTGTTAAATTTTATTTTTATATAATATTTTATTTTAATAACAGATTAATAGTGTTTAACTATTTGAGAGAGAGCAATAACAGGTCTGTGATGCCCTTAGATGTCCGGGGCTGCACGCGCGCTACAAT

>Haplotype_M

ATTGACAGATTGATAGCTCTTTCATGATTTAGTGGTTGGTGGTGCATGGCCGTTCTTAGTTCGTGGATATGATTTGTCTGGTTGATTCCGATAACGAGCGAGACTTTTATGTTATATTAAATAATATTATTTATAAATTTTATAGTTTTTATATAATATTTTATTTTAATAACAGATTAATAGTGTTTAACTATTTGAGAGAGAGCAATAACAGGTCTGTGATGCCCTTAGATGTCCGGGGCTGCACGCGCGCTACAAT

>Haplotype_N

ATTGACAGATTGATAGCTCTTTCATGATTTAGTGGTTGGTGGTGCATGGCCGTTCTTAGTTCGTGGATATGATTTGTCTGGTTGATTCCGATAACGAGCGAGACTTTTATGTTATATTAAATAATATTATTTATAAATTTTATATTTTATATAATATTTTATTTTAATAACAGATTAATAGTGCGTAACTA

>Haplotype_O

ATTGACAGATTGATAGCTCTTTCATGATTTAGTGGTTGGTGGTGCATGGCCGTTCTTAGTTCGTGGATATGATTTGTCTGGTTGATTCCGATAACGAGCGAGACTTTTATGTTATATTAAATAATATTATTGTTAAATTTTATGGTTTTATATAATATTTTATTTTAATAACAGATTAATAGTGTTTAACTATTTGAGAGAGAGCAATAACAGGTCTGTGATGCCCTTAGATGTCCGGGGCTGCACGCGCGCTACAAT

>Haplotype_P

ATTGACAGATTGATAGCTCTTTCATGATTTAGTGGTTGGTGGTGCATGGCCGTTCTTAGTTCGTGGATATGATTTGTCTGGTTGATTCCGATAACGAGCGAGACTTTTATGTTATATTAAATAATATTATTATTAAATTTTATTTTATATAATATTTTATTTTAATAACAGATTAATAGTGTTTAACTATTTGAGAGAGAGCAATAACAGGTCTGTGATGCCCTTAGATGTCCGGGGCTGCACGCGCGCTACAAT

>Haplotype_Q

ATTGACAGAGTGATAGCTCTTTCATGATTTAGTGGTTGGTGGTGCATGGCCGTTCTTAGTTCGTGGATATGATTTGTCTGGTTGATTCCGATAACGAGCGAGACTTTTATGTTATATTAAATAATATTATTTATAAATTTTATATATTATATAATATTTTATTTTAATAACAGATTAATAGTGTTTAACTATTTGAGAGAGAGCAATAACAGGTCTGTGA

>Haplotype_R

ATTGACAGATTGATAGCTCTTTCATGATTTAGTGGTTGGTGGTGCATGGCCGTTCTTAGTTCGTGGATATGATTTGTCTGGTTGATTCCGATAACGAGCGAGACTTTTATGTTATATTAAATAATATTATTATTAAATTTTATGGTTTTATATAATATTTTATTTTAATAACAGATTAATAGTGTTTAACTATTTGAGAGAGAGCAATAACAGGTCTGTGATGCCCTTAGATGTCCGGGGCTGCACGCGCGCTACAAT

>Haplotype_S

ATTGACAGATTGATAGCTCTTTCATGATTTAGTGGTTGGTGGTGCATGGCCGTTCTTAGTTCGTGGATATGATTTGTCTGGTTGATTCCGATAACGAGCGAGACTTTTATGTTATATTAAATAATATTATTTTAAATTTTATTTAATAATATTTTATTTTAATAACAGATTAATAGTGTTTAACTATTTGAGAGAGAGCAATAACAGGTCTGTGATGCCCTTAGATGTCCGGGGCTGCACGCGCGCTACAAT

>Haplotype_T

ATTGACAGATTGATAGCTCTTTCATGATTTAGTGGTTGGTGGTGCATGGCCGTTCTTAGTTCGTGGATATGATTTGTCTGGTTGATTCCGATAACGAGCGAGACTTTTATGTTATATTAAATAATATTATTATTAAATTTTATTTTTATATAATATTTTATTTTAATAACAGATTAATAGTGTTTAACTATTTGAGAGAGAGCAATAACAGGTCTGTGATGCCCTTAGATGTCCGGGGCTGCACGCGCGCTACAAT

>Haplotype_U

ATTGACAGATTGATAGCTCTTTCATGATTTAGTGGTTGGTGGTGCATGGCCGTTCTTAGTTCGTGGATATGATTTGTCTGGTTGATTCCGATAACGAGCGAGACTTTTATGTTATATTAAATAATATTATTTTAAATTTTATTTACATAATATTTTATTTTAATAACAGATTAATAGTGTTTAACTATTTGAGAGAGAGCAATAACAGGTCTGTGATGCCCTTAGATGTCCGGGGCTGCACGCGCGCTACAAT

# Appendix D. Fasta sequences of segmented haplotypes

>COXI_PART_I1_Hap_1_

GCTATTACTATGTTG

>COXI_PART_I1_Hap_2_

GCTATTACTATGTTA

>COXI_PART_I1_Hap_3_

GCTATTACTATATTA

>COXI_PART_I1_Hap_4_

GCTATTACTATATTG

>COXI_PART_I1_Hap_5_

GCCATTACTATGTTA

>COXI_PART_I1_Hap_6_

GCTATTACTATGCTT

>COXI_PART_I1_Hap_7_

GCTATTACTATACTT

>COXI_PART_I2_Hap_1_

TTATTTGATCGCAAT

>COXI_PART_I2_Hap_2_

TTATTTGATCGTAAT

>COXI_PART_I2_Hap_3_

TTGTTTGATCGTAAT

>COXI_PART_I2_Hap_4_

TTGGTTGATCGAAAT

>COXI_PART_I2_Hap_5_

TTATTCGATCGTAAT

>COXI_PART_I2_Hap_6_

TTAATTGATCGTAAT

>COXI_PART_I2_Hap_7_

TTGATTGATCGTAAT

>COXI_PART_I3_Hap_1_

TTTAATACTTCTTTC

>COXI_PART_I3_Hap_2_

TTTAATACTTCTTTT

>COXI_PART_I3_Hap_3_

TTTAATGGTTCTTTT

>COXI_PART_J1_Hap_1_

TTTGATCCTAGTGCT

>COXI_PART_J1_Hap_2_

TTTGACCCAAGTGCT

>COXI_PART_J1_Hap_3_

TTTGATCCAAGTGCT

>COXI_PART_J1_Hap_4_

TTTGATCCTAGTTCT

>COXI_PART_J1_Hap_5_

TTTGACCCAAGTGCC

>COXI_PART_J1_Hap_6_

TTTGATCCGAGTGCC

>COXI_PART_J1_Hap_7_

TTTGATCCTAGGGCT

>COXI_PART_J1_Hap_8_

TTTGATCCTAGAGCT

>COXI_PART_J1_Hap_9_

TTTGATCCTAGTTTT

>COXI_PART_J1_Hap_10_

TTTGATCCTAGTTTC

>COXI_PART_J1_Hap_11_

TTTGATCCTACGGGT

>COXI_PART_J1_Hap_12_

TTTGATCCTACAGGT

>COXI_PART_J1_Hap_13_

TTTGACCCTAGTGCT

>COXI_PART_J2_Hap_1_

GGTGGTAATCCTTTG

>COXI_PART_J2_Hap_2_

GGTGGTAATCCTCTT

>COXI_PART_J2_Hap_3_

GGTGGTAATCCTTTA

>COXI_PART_J2_Hap_4_

GGAGGTAATCCTTTG

>COXI_PART_J2_Hap_5_

GGGGGTAATCCTTTG

>COXI_PART_J2_Hap_6_

GGTGGTAATCCTCTG

>COXI_PART_J2_Hap_7_

GGTGGTAATCCATTG

>COXI_PART_J3_Hap_1_

ATTTATCAACATTTA

>COXI_PART_J3_Hap_2_

ATTTATCAACATTTG

>COXI_PART_J3_Hap_3_

ATTTATCAGCATTTA

>COXI_PART_J3_Hap_4_

ATTTATCAGCATTTG

>COXI_PART_J3_Hap_5_

GTTTATCAACATTTG

>COXI_PART_J3_Hap_6_

ATTTATCAACATCTT

>COXI_PART_K1_Hap_1_

TTTTGGTTCTTTGGT

>COXI_PART_K1_Hap_2_

TTCTGATTCTTTGGT

>COXI_PART_K1_Hap_3_

TTTTGATTCTTTGGA

>COXI_PART_K1_Hap_4_

TTTTGATTCTTTGGC

>COXI_PART_K1_Hap_5_

TTTTGGTTTTTTGGA

>COXI_PART_K1_Hap_6_

TTTTGGTTTTTTGGG

>COXI_PART_K1_Hap_7_

TTTTGGTTTTTTGGT

>COXI_PART_K1_Hap_8_

TTTTGGTTTTTTGGC

>COXI_PART_K1_Hap_9_

TTTTGATTTTTTGGT

>COXI_PART_K1_Hap_10_

TTTTGGTTTTTCGGT

>COXI_PART_K1_Hap_11_

TTTTGATTCTTTGGT

>COXI_PART_K2_Hap_1_

CATCCTGAGGTATAT

>COXI_PART_K2_Hap_2_

CATCCGGAAGTTTAT

>COXI_PART_K2_Hap_3_

CATCCAGAAGTTTAT

>COXI_PART_K2_Hap_4_

CATCCTGAGGTTTAT

>COXI_PART_K2_Hap_5_

CATCCTGAAGTTTAT

>COXI_PART_K2_Hap_6_

CATCCCGAAGTATAT

>COXI_PART_K2_Hap_7_

CATCCCGAGGTTTAT

>COXI_PART_K2_Hap_8_

CATCCTGAAGTATAT

>COXI_PART_K2_Hap_9_

CATCCTGAAGTGTAT

>COXI_PART_K2_Hap_10_

CATCCTGAGGTGTAT

>COXI_PART_K3_Hap_1_

ATTTTAATTCTTCCT

>COXI_PART_K3_Hap_2_

ATTTTGATTTTACCT

>COXI_PART_K3_Hap_3_

ATTTTGATTCTTCCT

>COXI_PART_K3_Hap_4_

ATCTTAATTCTTCCT

>COXI_PART_K3_Hap_5_

ATTTTAATTTTGCCT

>COXI_PART_K3_Hap_6_

ATTTTAATTTTACCT

>COXI_PART_L1_Hap_1_

GCTTTTGGTATTATT

>COXI_PART_L1_Hap_2_

GCTTTTGGAATTATT

>COXI_PART_L1_Hap_3_

GCTTTCGGTATTATT

>COXI_PART_L1_Hap_4_

GCTTTTGGAATTGTT

>COXI_PART_L1_Hap_5_

GCTTTTGGTATCATT

>COXI_PART_L2_Hap_1_

AGTCAAAGTACTCTT

>COXI_PART_L2_Hap_2_

AGTCAGTGTACTCTT

>COXI_PART_L2_Hap_3_

AGGCAAAGTACTCTT

>COXI_PART_L2_Hap_4_

AGTCAAAGTACTTTA

>COXI_PART_L2_Hap_5_

AGTCAAAGTACTCTC

>COXI_PART_L2_Hap_6_

AGTCAATGTACTTTA

>COXI_PART_L2_Hap_7_

AGTCAATGTACTTTG

>COXI_PART_L2_Hap_8_

AGTCAGTGTACTTTA

>COXI_PART_L2_Hap_9_

AGACAAAGTACTCTT

>COXI_PART_L2_Hap_10_

AGTCAATGTACCTTG

>COXI_PART_L3_Hap_1_

TATTTAACTGGTAAA

>COXI_PART_L3_Hap_2_

TATTTGACTGGTAAA

>COXI_PART_L3_Hap_3_

TATCTAACTGGTAAG

>COXI_PART_L3_Hap_4_

TATTTAACTGGTAAG

>COXI_PART_L3_Hap_5_

TATTTGACTGGTAAG

>COXI_PART_L3_Hap_6_

TATTTAACCGGTAAA

>COXI_PART_L3_Hap_7_

TACTTGACTGGTAAA

>COXI_PART_L3_Hap_8_

TATCTAACTGGTAAA

>COXI_PART_L3_Hap_9_

TACTTAACTGGTAAA

>COXI_PART_L3_Hap_10_

TATCTTACTGGTAAA

>COXI_PART_M1_Hap_1_

AAGGAGGTTTTTGGT

>COXI_PART_M1_Hap_2_

AAAGAAGTTTTTGGT

>COXI_PART_M1_Hap_3_

AAGGAAGTCTTTGGT

>COXI_PART_M1_Hap_4_

AAGGAGGTATTTGGT

>COXI_PART_M1_Hap_5_

AAGGAGGTGTTTGGT

>COXI_PART_M1_Hap_6_

AAAGAGGTGTTTGGT

>COXI_PART_M1_Hap_7_

AAGGAAGTATTTGGT

>COXI_PART_M1_Hap_8_

AAGGAAGTTTTTGGT

>COXI_PART_M1_Hap_9_

AAAGAGGTTTTTGGT

>COXI_PART_M1_Hap_10_

AAGGAAGTGTTTGGT

>COXI_PART_M1_Hap_11_

AAAGAGGTATTTGGT

>COXI_PART_M1_Hap_12_

AAGGAGGTCTTTGGT

>COXI_PART_M2_Hap_1_

ACTTTGGGTATGATT

>COXI_PART_M2_Hap_2_

ACTTTAGGTATGATT

>COXI_PART_M2_Hap_3_

ACCTTAGGTATAATT

>COXI_PART_M2_Hap_4_

ACCTTGGGTATAATT

>COXI_PART_M2_Hap_5_

ACTCTAGGTATGATT

>COXI_PART_M2_Hap_6_

TATCTTGGTATGGTT

>COXI_PART_M2_Hap_7_

ACTTTAGGTATAATT

>COXI_PART_M2_Hap_8_

ACTTTGGGTATAATT

>COXI_PART_M2_Hap_9_

TATTTAGGTATGGTT

>COXI_PART_M2_Hap_10_

TATTTGGGTATGGTT

>COXI_PART_M2_Hap_11_

TATTTAGGAATGGTT

>COXI_PART_M2_Hap_12_

ACTCTGGGTATGATT

>COXI_PART_M2_Hap_13_

ACTCTTGGTATGATT

>COXI_PART_M3_Hap_1_

TATGCTATTTTGAGA

>COXI_PART_M3_Hap_2_

TATGCTATTTTAAGA

>COXI_PART_M3_Hap_3_

TATGCTATTTTAAGT

>COXI_PART_M3_Hap_4_

TATGCAATTTTAAGT

>COXI_PART_M3_Hap_5_

TATGCGATTTTAAGT

>COXI_PART_M3_Hap_6_

TATGCAATTTTGAGT

>COXI_PART_M3_Hap_7_

TACGCGATTTTAAGT

>COXI_PART_M3_Hap_8_

TATGCTATTCTAAGT

>COXI_PART_M3_Hap_9_

TATGCTATTTTGAGT

>COXI_PART_M3_Hap_10_

TACGCTATCTTAAGT

>COXI_PART_M3_Hap_11_

TACGCTATTTTAAGT

>COXI_PART_M3_Hap_12_

TATGCTATTTTAAGG

>COXI_PART_M3_Hap_13_

TACGCTATTTTAAGA

>COXI_PART_M3_Hap_14_

TATGCTATTCTAAGA

>COXI_PART_M3_Hap_15_

TATGCTATTCTTAGA

>COXI_PART_N1_Hap_1_

ATTGGTTTAATTGGT

>COXI_PART_N1_Hap_2_

ATTGGTTTGATTGGT

>COXI_PART_N1_Hap_3_

ATTGGTTTAATCGGT

>COXI_PART_N1_Hap_4_

ATTGGATTGATTGGT

>COXI_PART_N1_Hap_5_

ATTGGATTAATTGGT

>COXI_PART_N1_Hap_6_

ATTGGGTTAATTGGT

>COXI_PART_N2_Hap_1_

TGTGTTGTTTGGGCT

>COXI_PART_N2_Hap_2_

TGTGTAGTATGGGCT

>COXI_PART_N2_Hap_3_

TGTGTAGTGTGGGCT

>COXI_PART_N2_Hap_4_

TGTGTAGTTTGAGCT

>COXI_PART_N2_Hap_5_

TGTGTAGTTTGGGCT

>COXI_PART_N2_Hap_6_

TGTGTTGTCTGGGCT

>COXI_PART_N2_Hap_7_

TGTGTTGTTTGAGCT

>COXI_PART_N2_Hap_8_

TGTGTTGTTTGGGCC

>COXI_PART_N2_Hap_9_

TGTGTGGTTTGGGCT

>COXI_PART_N3_Hap_1_

CATCACATGTATACT

>COXI_PART_N3_Hap_2_

CATCACATATATACT

>COXI_PART_N3_Hap_3_

CATCATATGTATACT

>COXI_PART_N3_Hap_4_

CATCATATATATACT

>COXI_PART_N3_Hap_5_

CATCATATGTATACC

>COXI_PART_N3_Hap_6_

CACCATATGTATACT

>COXI_PART_N3_Hap_7_

CATCATATGTACACT

>COXI_PART_O1_Hap_1_

GTTGGTATGGATATT

>COXI_PART_O1_Hap_2_

GTTGGTATAGATTTT

>COXI_PART_O1_Hap_3_

GTTGGTATGGATTTT

>COXI_PART_O1_Hap_4_

GTTGGTATGGATTTC

>COXI_PART_O1_Hap_5_

GTTGGTATGGATATC

>COXI_PART_O1_Hap_6_

GTTGGGATGGATATT

>COXI_PART_O1_Hap_7_

GTTGGTATAGATATT

>COXI_PART_O1_Hap_8_

GTTGGTATGGACATT

>COXI_PART_O1_Hap_9_

GTTGGAATGGATTTT

>COXI_PART_O1_Hap_10_

GTTGGAATGGATTTC

>COXI_PART_O1_Hap_11_

GTGGGTATAGATATT

>COXI_PART_O1_Hap_12_

GTTGGTATATATTTT

>COXI_PART_O1_Hap_13_

GTAGGTATGGATATT

>COXI_PART_O1_Hap_14_

GTTGGAATGGATATT

>HVR_I_PART_A_Hap_1_

GTTGGATAACTGAGGTAATTCTTGAGCTAATACACGCTACCTATACCACATTAGTGGTGCGTTTATTTGATTAAACCATTATTTTGGTTGACTCAAAAT

>HVR_I_PART_A_Hap_2_

GTTGGATAACTGAGGTAATTCTTGAGCTAATACACGCTATTTATACCACATTAGTGGTGCGTTTATTTGATTAAACCATTATTTTGGTTGACTCAAAAT

>HVR_I_PART_A_Hap_3_

GTTGGATAACTGACGTAATTCTTGAGCTAATACACGCTATTTATACCACATTAGTGGTGCGTTTATTTGATTAAACCATTATAACGGTTGACTCAAAAT

>HVR_I_PART_A_Hap_4_

GTTGGATAACTGAGGTAATTCTTGAGCTAATACACGCTATTTATACCACATTAGTGGTGCGTTTATTTGATTAAACCATTATAACGGTTGACTCAAAAT

>HVR_I_PART_A_Hap_5_

GTTGGATAACTGAGGTAATTCTTGAGCTAATACACGCTATTTATACCACATTAGTGGTGCGTTTATTTGATTAAACCATTTTAACGGTTGACTCAAAAT

>HVR_I_PART_A_Hap_6_

GTTGGATAACTGAGGTAATTCTTGAGCTAATACACGCTGTTAATACCACATTAGTGGTGCGTTTATTTGATTAAACCATTATATTGGTTGACTCAAAAT

>HVR_I_PART_A_Hap_7_

GTTGGATAACTGAGGTAATTCTTGAGCTAATACACGCTATTTATACCACATTAGTGGTGCGTTTATTTGATTAAACCATTTATACTGGTTGACTCAAAAT

>HVR_I_PART_A_Hap_8_

GTTGGATAACTGAGGTAATTCTTGAGCTAATACACGCTATTTATACCACATTAGTGGTGCGTTTATTTGATTAAACCATTTTATACTGGTTGACTCAAAAT

>HVR_I_PART_A_Hap_9_

GTTGGATAACTGAGGTAATTCTTGAGCTAATACACGCTATTTATACCACATTAGTGGTGCGTTTATTTGATTAAACCATTTTTATATTGGTTGACTCAAAAT

>HVR_I_PART_A_Hap_10_

GTTGGATAACTGAGGTAATTCTTGAGCTAATACACGCTATTTATACCACATTAGTGGTGCGTTTATTTGATTAAACCATTTTATATTGGTTGACTCAAAAT

>HVR_I_PART_A_Hap_11_

GTTGGATAACTGAGGTAATTCTTGAGCTAATACACGCTATTTATACCACATTAGTGGTGCGTTTATTTGATTAAACCATTTTTATATATTGGTTGACTCAAAAT

>HVR_I_PART_A_Hap_12_

GTTGGATAACTGAGGTAATTCTTGAGCTAATACACGCTATTTATACCACATTAGTGGTGCGTTTATTTGATTAAACCATTTATATTGGTTGACTCAAAAT

>HVR_I_PART_A_Hap_13_

GTTGGATAACTGAGGTAATTCTTGAGCTAATACACGCTATTTATACCACATTAGTGGTGCGTTTATTTGATTAAACCATTATATAACGGTTGACTCAAAAT

>HVR_I_PART_B_Hap_1_

ATCCTCGCTGATTTTGTTACTAAAACATACCGTATGTGTATCTGGTTTATCAACTTTCGATGGTAGGGTATTGGCCTACCATGGTTGTGACGGATAACGGAGAATTAGGGT

>HVR_I_PART_B_Hap_2_

ATCCTCGCTGATTTTGTTACAAAAACATACCGTATGTGTATCTGGTTTATCAACTTTCGATGGTAGGGTATTGGCCTACCATGGTTGTGACGGATAACGGAGAATTAGGGT

>HVR_I_PART_B_Hap_3_

ATCCTTGCTGATTTTGTTACTAAAACATACCGTATGTGTATCTGGTTTATCAACTTTCGATGGTAGGGTATTGGCCTACCATGGTTGTGACGGATAACGGAGAATTAGGGT

>HVR_I_PART_C_Hap_1_

TCGACTCCGGAGAGGGAGCCTGAGAAACGGCTACCACATCCAAGGAAGGCAGCAGGCGCGAAAATTACCCAATTTTAGTTTAAAGAGGTAGTGACGAAAAATGACAACCA

>HVR_I_PART_C_Hap_2_

TCGACTCCGGAGAGGGAGCCTGAGAAACGGCTACCACATCCAAGGAAGGCAGCAGGCGCGAAAATTACCCAATTTTAGTTCAAAGAGGTAGTGACGAAAAATGACAACCA

>HVR_I_PART_C_Hap_3_

TCGACTCCGGAGAGGGAGCCTGAGAAACGGCTACCACATCCAAGGAAGGCAGCAGGCGCGAAAATTACCCAATTTTAGTTAAAAGAGGTAGTGACGAAAAATGACAACCA

>HVR_I_PART_C_Hap_4_

TCGACTCCGGAGAGGGAGCCTGAGAAATGGCTACCACATCCAAGGAAGGCAGCAGGCGCGAAAATTACCCAATTTTAGTTAAAAGAGGTAGTGACGAAAAATGACAACCA

>HVR_I_PART_D_Hap_1_

AATATTATTATTAATATTTGGATTGAAAATCTTCAAGTTTAAATAACTTGTTGGTAAAGGAAAGGGCAAGTCTGGTGCCAGCAGCCGCGGTAATACCAGC

>HVR_I_PART_D_Hap_2_

AATATTATTATTAATATTTGGATTGAAAATCTTCAAGTTTAAATAACTTGTTGGTAAAGGTTTGGGCAAGTCTGGTGCCAGCAGCCGCGGTAATACCAGC

>HVR_I_PART_D_Hap_3_

AATATTATTATTAATATTTGGATTGAAAATCTTCAAGTATAAATAACTTGTTGGTAAAGGAAAGGGCAAGTCTGGTGCCAGCAGCCGCGGTAATACCAGC

>HVR_I_PART_D_Hap_4_

AATATTATTATATTAATATTTGGATTGAAAATCTTCAAGTTTAAATAACTTGTTGGTAAAGGAAAGGGCAAGTCTGGTGCCAGCAGCCGCGGTAATACCAGC

>HVR_4_PART_A_Hap_1_

ATTGACAGATTGATAGCTCTTTCATGATTTAGTGGTTGGTGGTGCATGGCCGTTCTTAGTTCGTGGATATGATTTGTCTGGTTGATT

>HVR_4_PART_A_Hap_2_

ATTGACAGAGTGATAGCTCTTTCATGATTTAGTGGTTGGTGGTGCATGGCCGTTCTTAGTTCGTGGATATGATTTGTCTGGTTGATT

>HVR_4_PART_A_Hap_3_

ATTGACAGATTGATAGCTCTTTCATGATCTAGTGGTTGGTGGTGCATGGCCGTTCTTAGTTCGTGGATATGATTTGTCTGGTTGATT

>HVR_4_PART_B_Hap_1_

CCGATAACGAGCGAGACTTTTATGTTATATTAAATAATATTATTTATAAATTTTATATTTTATATAATATTTTATTTTAATAACA

>HVR_4_PART_B_Hap_2_

CCGATAACGAGCGAGACTTTTATGTTATATTAAATAATATTATTTATAAATTTTATATATTATATAATATTTTATTTTAATAACA

>HVR_4_PART_B_Hap_3_

CCGATAACGAGCGAGACTTTTATGTTATATTAAATAATATTATTATTAAATTTTATTTTTATATAATATTTTATTTTAATAACA

>HVR_4_PART_B_Hap_4_

CCGATAACGAGCGAGACTTTTATGTTATATTAAATAATATTATTATTAAATTTTATTTTATATAATATTTTATTTTAATAACA

>HVR_4_PART_B_Hap_5_

CCGATAACGAGCGAGACTTTTATGTTATATTAAATAATATTATTATTAAATTTTATGGTTTTATATAATATTTTATTTTAATAACA

>HVR_4_PART_B_Hap_6_

CCGATAACGAGCGAGACTTTTATGTTATATTAAATAATATTATTGTTAAATTTTATGGTTTTATATAATATTTTATTTTAATAACA

>HVR_4_PART_B_Hap_7_

CCGATAACGAGCGAGACTTTTATGTTATATTAAATAATATTATTGTTAAATTTTATTTTTATATAATATTTTATTTTAATAACA

>HVR_4_PART_B_Hap_9_

CCGATAACGAGCGAGACTTTTATGTTATATTAAATAATATTATTTATAAATTTTATAGTTTTTATATAATATTTTATTTTAATAACA

>HVR_4_PART_B_Hap_8_

CCGATAACGAGCGAGACTTTTATGTTATATTAAATATAATTATTTTGTTTATTTTAATATAAATAATTAATATTTTAATAACA

>HVR_4_PART_B_Hap_10_

CCGATAACGAGCGAGACTTTTATGTTATATTAAATAATATTATTTTAAATTTTATTTAATAATATTTTATTTTAATAACA

>HVR_4_PART_B_Hap_11_

CCGATAACGAGCGAGACTTTTATGTTATATTAAATATTATTATTTTATTATATAAATAATAATTATTTTAATAACA

>HVR_4_PART_B_Hap_12_

CCGATAACGAGCGAGACTTTTATGTTATATTAAATATTATTATTTTTTATATAAATAATAATTATTTTAATAACA

>HVR_4_PART_B_Hap_13_

CCGATAACGAGCGAGACTTTTATGTTATATTAAATATTATTATTTTATTATTTTATATAAATAATTAATATTTTAATAACA

>HVR_4_PART_B_Hap_14_

CCGATAACGAGCGAGACTTTTATGTTATATTAAATATTATTATTTGTTTATTTTTTATATAAATAATTAATATTTTAATAACA

>HVR_4_PART_B_Hap_15_

CCGATAACGAGCGAGACTTTTATGTTATATTAAATATTATTATTTGTTTATTTTATATAAATAATTAATATTTTAATAACA

>HVR_4_PART_B_Hap_16_

CCGATAACGAGCGAGACTTTTATGTTATATTAAATATTATTATTTGTTTATTTTAATATAAATAATTAATATTTTAATAACA

>HVR_4_PART_B_Hap_17_

CCGATAACGAGCGAGACTTTTATGTTATATTAAATATTATTATTTTGTTTATTTTAATATAAATAATTAATATTTTAATAACA

>HVR_4_PART_B_Hap_18_

CCGATAACGAGCGAGACTTTTATGTTATATTAAATAATATTATTTTAAATTTTATTTACATAATATTTTATTTTAATAACA

>HVR_4_PART_C_Hap_1_

GATTAATAGTGTTTAACTATTTGAGAGAGAGCAATAACAGGTCTGTGATGCCCTTAGATGTCCGGGGCTGCACGCGCGCTACAAT

>HVR_4_PART_C_Hap_2_

GATTAATAGTGTTTAACTATTTGAGAGAGAGCGATAACGGGTCTGTGATGCCCTTAGATGTCCGGGGCTGCACGCGCGCTACAAT

>HVR_4_PART_C_Hap_3_

GATTAATAGTGTTTAACTATTTGAGAGAGAGCGATAACAGGTCTGTGATGCCCTTAGATGTCCGGGGCTGCACGCGCGCTACAAT

>HVR_4_PART_C_Hap_4_

GATTAATAGTGTTTAACTATTTGAGAGAGAGCGATAACAGGTATGTGATGCCCTTAGATGTCCGGGGCTGCACGCGCGCTACAAT
