# Supplementary figures and images for: Genetic diversity within Strongyloides fuelleborni: mitochondrial genome analysis reveals a clear African and Asian division
Source: Parasitology. 2025 Jun 23;152(7):735–44. doi: 10.1017/S0031182025100243 (PMC12418279; doi:10.1017/S0031182025100243)

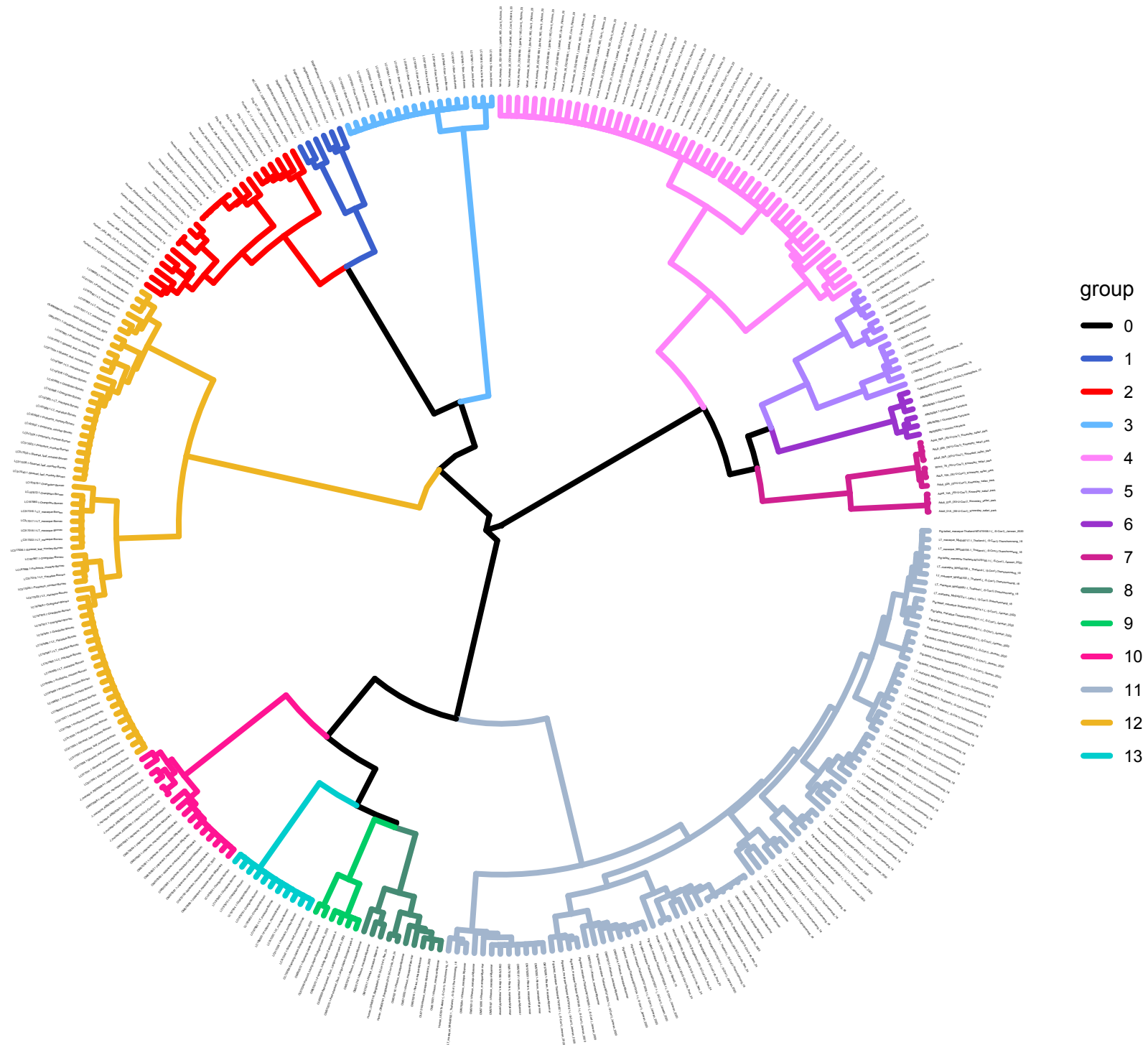

Supplement: Richins et al. supplementary material [file S0031182025100243sup001.zip › S0031182025100243sup001/File_S3.Rplot_WARDS_tree_Supp_File_S3.pdf]

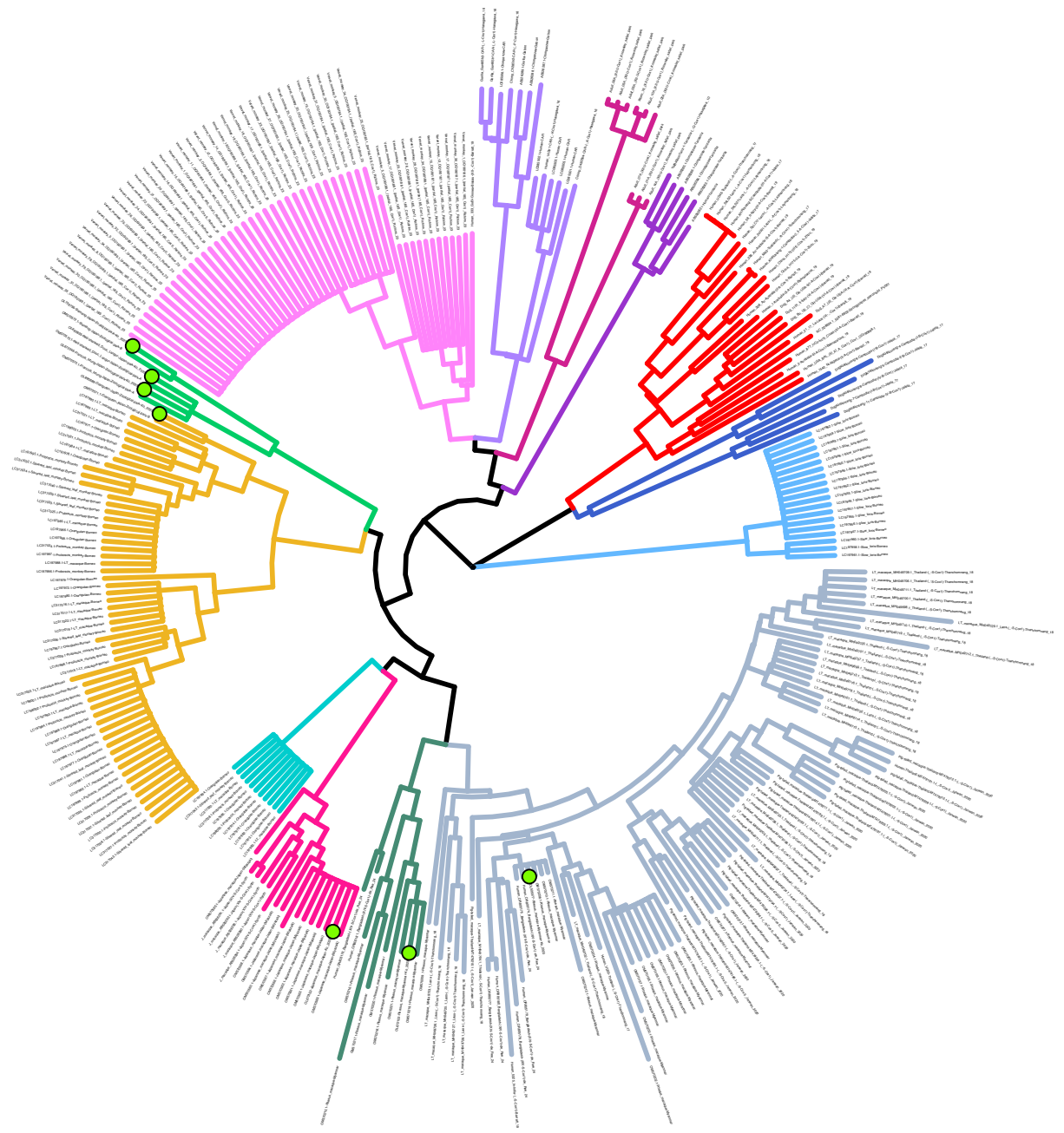

- group
- 0
  - 1
  - 2
  - 3
  - 4
  - 5
  - 6
  - 7
  - 8
  - 9
  - 10
  - 11
  - 12
  - 13

Supplement: Richins et al. supplementary material [file S0031182025100243sup001.zip › S0031182025100243sup001/File_S4.Rplot_Supp_file_NJ_Supp_file_S4.pdf]
